# One Shock, Not One Cure: Electroporation Reveals Disease-Specific Constraints in Hepatocyte Gene Editing Therapy

**DOI:** 10.3390/biology14081091

**Published:** 2025-08-20

**Authors:** Callie Clark, Menam Pokhrel, Benjamin Arthur, Pramita Suresh, Ilayda Ates, Justin Gibson, Abishek Dhungana, Ryan Mehlem, Andrew Boysia, Mugdha V. Padalkar, Achala Pokhrel, Jing Echesabal-Chen, Anne Vonada, Alexis Stamatikos, Olga V. Savinova, Markus Grompe, Renee N. Cottle

**Affiliations:** 1 Department of Bioengineering, Clemson University, Clemson, SC 29634, USA; calliedclark@gmail.com (C.C.); mpokhre@clemson.edu (M.P.); bbarthu@clemson.edu (B.A.); pramitasuresh@icloud.com (P.S.); ilayda.ates9@gmail.com (I.A.); jrg6@g.clemson.edu (J.G.); adhunga@clemson.edu (A.D.); rmehlem@clemson.edu (R.M.); aboysia@g.clemson.edu (A.B.); 2 Department of Biomedical Sciences, New York Institute of Technology College of Osteopathic Medicine, Old Westbury, NY 11568, USA; mpadalka@nyit.edu (M.V.P.); osavinov@nyit.edu (O.V.S.); 3 Department of Food, Nutrition, and Packaging Sciences, Clemson University, Clemson, SC 29634, USA; achalap@clemson.edu (A.P.); jchen11@clemson.edu (J.E.-C.); adstama@clemson.edu (A.S.); 4 Papé Family Pediatric Research Institute, Oregon Health & Science University, Portland, OR 97239, USA; vonada@ohsu.edu (A.V.); grompem@ohsu.edu (M.G.); 5 Center for Human Genetics, Clemson University, Greenwood, SC 29646, USA

**Keywords:** CRISPR-Cas9, gene editing, hepatocyte transplantation, electroporation, liver research, familial hypercholesterolemia, atherosclerosis

## Abstract

We previously demonstrated lipid nanoparticle-mediated CRISPR-Cas9 gene editing to disrupt the gene encoding cytochrome P450 oxidoreductase (Cypor), combined with transient administration of acetaminophen (APAP), to repopulate the liver with healthy hepatocytes and rescue a phenylketonuria mouse model. This study aimed to investigate electroporation-mediated delivery of *Cypor*-targeting CRISPR-Cas9 ribonucleoproteins into wild-type hepatocytes, combined with liver engraftment under APAP treatment, as an in vivo selection approach in a mouse model of homozygous familial hypercholesterolemia (*Ldlr*^−/−^). Electroporation provides higher delivery efficiency compared to lipid nanoparticles. We observed engraftment levels up to 13% engraftment of electroporated Cypor-deficient hepatocytes with indels in the liver of *Ldlr*^−/−^ mice after transient APAP administration, while negligible engraftment was observed in no-APAP controls (mean 9% and 2%, respectively, *p* = 0.0121). The engraftment of Cypor-deficient *Ldlr*^+/+^ hepatocytes was associated with reductions in LDL-cholesterol (18%) and triglycerides (52%) compared to the untransplanted control *Ldlr*^−/−^ mice fed a Western diet for 5 weeks, but offered no protection from the development of diet-induced aortic root atherosclerosis or liver steatosis. While biochemical markers for liver damage normalized after discontinuation of APAP, we observed persistent lipid accumulation in the liver of *Ldlr*^−/−^ mice grafted with Cypor-deficient *Ldlr*^+/+^ hepatocytes, likely stemming from the impact of Cypor deficiency on cholesterol clearance. Therefore, the combination of CRISPR-Cas9-mediated Cypor knockdown to induce clonal expansion of gene-edited hepatocytes using transient APAP administration is not a viable therapeutic strategy for familial hypercholesterolemia due to the essential role of Cypor in cholesterol metabolism. Unlike findings from phenylketonuria mouse model studies, the loss of Cypor function could not be compensated by unedited native hepatocytes in *Ldlr*^−/−^ mice. Collectively, our results demonstrate that electroporation is a viable and informative approach for evaluating gene editing strategies for the treatment of inherited metabolic diseases that affect the liver. Our electroporation procedure revealed that a one-size-fits-all gene editing strategy may not be universally applicable for treating inherited metabolic liver disorders. Tailored gene editing and selection strategies may be needed for different liver disorders.

## Simple Summary

Liver transplantation remains the only definitive treatment for inherited metabolic liver diseases, but it carries significant drawbacks. In earlier research, we showed that we could use a gene-editing tool called CRISPR-Cas9 to engineer liver cells (called hepatocytes) and then help those edited hepatocytes to expand in the liver using short-term treatment with the common pain reliever acetaminophen (APAP). This cell therapy approach successfully cured a mouse model of phenylketonuria, a rare genetic disease of the liver. Here, we applied this approach to a different genetic liver disease, familial hypercholesterolemia, which is characterized by high cholesterol levels and increased risks of developing atherosclerotic cardiovascular disease. We gene-edited healthy liver hepatocytes and transplanted them into mice with the disease, then used APAP to select for the gene-edited hepatocytes in the liver. The gene-edited hepatocytes engrafted up to 13% of liver hepatocytes and the lipid levels decreased. However, disabling the gene (Cypor) using CRISPR-Cas9 for APAP selection also caused problems with how fats were processed in the liver. Collectively, our results suggest that while our cell-based gene editing approach can work well in some liver diseases, it might not be the best fit for all of them.

## 1. Introduction

Homozygous familial hypercholesterolemia (HoFH) is a life-threatening autosomal co-dominant disorder characterized by elevated plasma cholesterol levels (>500 mg/dL), leading to atherosclerotic cardiovascular disease [1]. Although a small proportion of HoFH cases is caused by mutations in apolipoprotein B (APOB), proprotein convertase subtilisin/kexin type 9 (PCSK9), and low-density lipoprotein receptor adaptor protein 1 (LDLRAP1) [2], the most predominant form of HoFH is attributed to the inheritance of two copies of low-density lipoprotein receptor (LDLR) null mutant genes [3]. Affected patients have 3–6 times higher LDL-cholesterol (LDL-C) levels in the blood [4], 100 times enhanced risk for developing early atherosclerotic cardiovascular disease compared to the general population, and higher incidence of mortality by the third decade of life when left untreated [5].

Despite a significant rise in cases over the years (about 1 in 160,000 to 300,000 having HoFH) [6], life-long lipid-lowering therapies such as statins are the standard treatment for patients with HoFH diagnosed in early childhood [7]. However, response to statin treatments is variable in the general population of familial hypercholesterolemia patients, with the least response in HoFH patients associated with a slight reduction in LDL-C plasma levels by 10–25%. Further reduction in LDL-C levels by 10–15% can be achieved by combining statins with the cholesterol absorption inhibitor ezetimibe [1,8]. Although providing enhanced survival, lipid-lowering therapy fails to achieve LDL-C target levels and is further limited by tolerability and efficacy issues [1,9]. LDL-C apheresis is the standard treatment for patients with low tolerance for drug therapy, but levels of LDL-C are not maintained and can rebound rapidly within days after treatment [10]. Similarly, use of liver allografts from healthy donors, either alone or with a heart transplant, in severe cases, is effective in correcting total cholesterol and LDL-C levels; however, this procedure has a high risk of mortality from complications due to the procedure and lifelong treatment with immunosuppressive therapy [11,12,13]. The challenges associated with existing treatment of HoFH provided the impetus to develop novel therapeutic approaches for HoFH and other liver-based metabolic disorders.

As an alternative to liver transplantation, ex vivo gene therapy was used to treat HoFH in the first clinical trial (NCT00004809), conducted over 30 years ago, involving viral-mediated gene addition of the functional *LDLR* into autologous hepatocytes isolated from the patient’s resected liver and infusion back into the patient [14]. The approach was unsuccessful at sufficiently reducing lipid levels, with patients continuing to have elevated unhealthy LDL-C levels at the end of treatment [15], which was attributed to low gene transfer efficiency or insufficient engraftment level [16]. With the discovery of CRISPR-Cas9 and improved protocols for precise gene editing, significant improvements have been made to gene transfer efficiency. Nonetheless, the problem associated with in vivo hepatocyte engraftment still prevails because mature hepatocytes only undergo proliferation to replace damaged hepatocytes in the event of cellular injury [17,18]. Thus, donor cells must have a selective advantage over native hepatocytes to undergo proliferation and repopulate the liver. The criterion for selective advantage is autonomous cell injury that does not impact neighboring hepatocytes and promotes liver regeneration. Since healthy hepatocytes have a selective advantage in only a limited number of disorders, such as hereditary tyrosinemia type 1 (HT1), Wilson’s disease, and methylmalonic acidemia, there is a critical need for strategies that introduce selective pressure to promote the expansion of donor hepatocytes in the liver. Such approaches are critical for the advancement of hepatocyte transplantation as a viable cell therapy for a broader range of inherited metabolic liver diseases, including HoFH [17].

Acetaminophen (APAP) is a commonly available over-the-counter analgesic and antipyretic [19]. APAP metabolism is catalyzed by CYP enzymes in the presence of cofactor, cytochrome P450 oxidoreductase (Cypor), into a reactive metabolite, NAPQI [20,21]. While safe at low doses, APAP is associated with liver toxicity at high doses, leading to acute liver injury and both fatal and non-fatal hepatic necrosis [22]. However, this toxicity can be harnessed to induce controlled hepatic injury, introducing a selective advantage for healthy donor hepatocytes to proliferate and repopulate the liver following hepatocyte transplantation. Engraftment of healthy hepatocytes corresponding to 5–10% is the therapeutic threshold for various liver-based disorders caused by single gene defects [23]. We have shown that transient administration of APAP activates the clonal expansion of Cypor-deficient donor hepatocytes, repopulating up to 50% of the liver mass and rescuing a phenylketonuria (PKU) mouse model [24,25]. Building on our preliminary studies demonstrating electroporation-mediated delivery of CRISPR-Cas9 into hepatocytes to rescue a fumarylacetoacetate hydrolase (Fah) knockout mouse model of HT1 and APAP-mediated selection of hepatocytes [26,27,28], we employed an ex vivo gene editing approach to transplant gene-edited hepatocytes, isolated from healthy wild-type C57BL/6J mice, into the *Ldlr*^−/−^ mouse model of HoFH. We show that transient APAP administration provided Cypor-deficient *Ldlr*^+/+^ hepatocytes a selective advantage to expand in the liver to levels within the desired threshold for expected clinical benefit, resulting in reduced lipid levels, but not providing protective effects against atherosclerosis development in *Ldlr*^−/−^ mice.

## 2. Materials and Methods

### 2.1. Animals and Animal Care

All mice received humane care in compliance with the Institutional Animal Care and Use Committee regulations of Clemson University. Donor hepatocytes were isolated from wild-type male C57BL/6J mice, 8–10 weeks old, maintained on a regular chow diet (2018 Teklad Global 18% Protein Rodent Diet, Inotiv, Madison, WI, USA). Male C57BL/6J *Fah*∆exon5 mice (*Fah*^−/−^) containing a 105 bp deletion in exon 5 of the fumarylacetoacetate hydrolase gene, 8–10 weeks old, were used as control recipients of hepatocyte transplantation to validate the engraftment potential of gene-edited hepatocytes. *Fah*^−/−^ mice were maintained on a high-energy chow diet (5LJ5, PicoLab, Richmond, IN, USA) and given water supplemented with 2-(2-nitro-4-trifluoro-methylbenzyol)-1,3 cyclohexanedione (NTBC) at a concentration of 8 mg/mL (A384235, Ambeed, Arlington Heights, IL, USA). Male B6.129S7-*Ldlr*^tm1Her^/J mice (*Ldlr*^−/−^), homozygous for the *Ldlr^tm1Her^* mutation (002207, The Jackson Laboratory, Bar Harbor, ME, USA), 6 weeks old, were used as recipients of hepatocyte transplantation to assess the effects of engraftment on lipid levels and the progression of atherosclerosis in combination with our cell therapy approach. *Ldlr*^−/−^ mice were maintained on a regular chow (5LOD, PicoLab, Richmond, IN, USA) diet during the APAP selection period and then switched to a Western atherogenic diet (TD. 88137, Envigo Bioproducts, Madison, WI, USA). To establish the APAP selection procedure, C57BL/6J males, 6–10 weeks old, were used as recipients. *Ldlr*^−/−^ mice were sacrificed at 10 days following APAP selection or after 12 weeks on a Western diet.

### 2.2. Hepatocyte Electroporation and Transplantation

Hepatocytes were isolated from anesthetized mice using a three-step perfusion described in [27,29] or using the gentleMACS Liver Perfusion Kit (130-128-030, Miltenyi Biotec, Auburn, CA, USA) for mouse and rat hepatocytes on a gentleMACS Dissociator following the manufacturer’s protocol. Following isolation, hepatocyte viability was quantified by trypan blue staining using a hemocytometer. Hepatocytes with a viability > 70% were used in electroporation experiments immediately after washing steps in cold HMX media. HMX media consisted of DMEM high glucose with GlutaMAX (11995065, Thermo Fisher Scientific, Waltham, MA, USA), 10% fetal bovine serum (F2442, Sigma-Aldrich, St. Louis, MO, USA), 100 U/mL penicillin, 0.1 mg/mL streptomycin (30002CI, Corning, Corning, NY, USA), and 10 mM HEPES (BP299-1, Fisher Scientific, Waltham, MA, USA).

Freshly isolated hepatocytes were electroporated using a Lonza 4D-Nucleofector X Unit using the program CM-150 under the following conditions: 100 μL P3 Primary Cell 4D-Nucleofector solution (V4XP-3024, Lonza, Bend, OR, USA), 1.2 × 10^6^ cells; 1.5 μL 20 g/L *Cypor*-sgRNA (IDT); 4.9 μL of 61 μM V3 SpCas9 (1081059, Integrated DNA Technologies, Coralville, IA, USA). The *Cypor*-sgRNA contained the same guide sequence as used in [25]. Immediately after electroporation, cytokine recovery media was added to hepatocytes for transient incubation to enhance viability as described in [28]. Electroporated cells were also plated as described in [27]. A 0.25 g/L Corning Matrigel basement membrane matrix (356234, Corning, Corning, NY, USA) was added as an overlay to each well at 24 h after plating. Plated cells were imaged 24 h after plating. Hepatocyte viability was quantified 24 h after plating using CyQUANT MTT Cell Viability Assay (V13154, Thermo Fisher Scientific, Waltham, MA, USA). Genomic DNA isolated from plated cells and homogenized liver tissue as described in [27,28], respectively, were assessed for on-target gene editing efficiency. Cas9 insertions and deletions (indels) were quantified by tracking of indels by decomposition [30] and Synthego Inference of CRISPR Edits [31].

Electroporated hepatocytes were intrasplenically injected as described in [28]. Briefly, for each recipient, 500,000 viable cells were resuspended in 120 µL room temperature HMX medium, followed by injection into recipient mice, within 2 h after electroporation. Transplanted *Fah*^−/−^ control mice immediately underwent periods off and on NTBC water until the weights stabilized off-NTBC water as described in [28]. *Fah*^−/−^ mice lack the terminal enzyme of the tyrosine catabolism pathway and develop progressive liver disease. Treatment with NTBC prevents the accumulation of hepatotoxic metabolites and rescues the phenotype in *Fah*^−/−^. This model is widely used as a recipient strain in therapeutic liver repopulation studies because any hepatocytes expressing Fah have a strong selective advantage to expand when NTBC treatment is removed [32].

### 2.3. In Vitro LDL-C Uptake Assay

Plated wild-type (WT), Cypor-deficient, and Ldlr-deficient hepatocytes were treated with LDL-C from human plasma labeled with pHrodo Red (L34356, Fisher Scientific, Waltham, MA, USA) according to the manufacturer’s protocol. Cells were incubated in HMX media supplemented with 10% lipid-depleted fetal bovine serum (CFB-50, Omega Scientific, Tarzana, CA, USA) for 12 h followed by the addition of 10 μg/mL pHrodo Red-LDL for 4 h. Cells were washed three times using DPBS. Cells were dissociated using 0.25% trypsin-EDTA (25200072, Thermo Fisher Scientific, Waltham, MA, USA) at 37 °C for 5 min. Dissociated cells were washed and analyzed for uptake using a Cytoflex S flow cytometer (Beckman Coulter, Indianapolis, IN, USA).

### 2.4. APAP Selection

Starting 12 days after transplantation, APAP was administered twice per week via intraperitoneal injections (IP) as described in [25] to activate the selection of gene-edited Cypor-deficient hepatocytes. Briefly, the APAP dose was started at 200 mg/kg (C57BL/6J tail vein injection) or 225 mg/kg (C57BL/6J and *Ldlr*^−/−^ transplant) and increased by 10–25 mg/kg increments until two consecutive elevated alanine aminotransferase (ALT) measurements (≥1000 U/L) were observed. At 6 h after APAP injection, mice were anesthetized under isoflurane for peripheral blood collection. The ALT level was quantified in plasma using the ALT Colorimetric kit (A526-120, Teco Diagnostics, Anaheim, CA, USA) with modifications to the manufacturer’s protocol for ALT quantification in plasma.

### 2.5. Histology and Immunofluorescence Staining

Hematoxylin and eosin (H&E) staining and Fah immunohistochemistry (IHC) staining were performed as described in [28]. Liver tissues were sliced into 3 mm segments and fixed in 10% neutral-buffered formalin (HT501640, Millipore Sigma, St. Louis, MO, USA) at room temperature for 24 h. The fixed segments were then embedded in paraffin and sectioned. For Cypor immunofluorescence staining (IF), liver sections were deparaffinized by heat-induced epitope retrieval in Tris EDTA Buffer (ab93684, Abcam, Waltham, MA, USA). Slides were blocked in 0.15 M glycine (AAA1381636, Fisher Scientific, Waltham, MA, USA) in PBS with 10% normal goat serum (5560-0007, SeraCare, Milford, MA, USA). Samples were incubated overnight at 4 °C with rabbit anti-Cypor (ab180597, Abcam, Waltham, MA, USA) at a 1:200 dilution in 2% normal goat serum in PBS. Samples were washed three times for 5 min in PBS followed by 1 h incubation of secondary antibody, Alexa 555 goat anti-rabbit IgG (A27039, Thermo Fisher Scientific, Waltham, MA, USA), at a 1:500 dilution in 2% normal goat serum in PBS. Slides were washed three times for 5 min in PBS and stained with Hoechst 33342 (H3570, Fisher Scientific, Waltham, MA, USA) at a 1:10000 dilution in PBS. Coverslips were mounted on slides with Fluoromount (00-4958-02, Thermo Fisher Scientific, Waltham, MA, USA). Imaging was performed on a Leica Laser Microdissection with a Leica DFC7000T camera (Leica Camera, Wetzlar, Germany). The percentage of Fah-positive area was quantified using at least three IHC liver sections stained against Fah using ImageJ bundled with Java 8 software version 1.54g (Rasband, W.S., ImageJ, US National Institutes of Health, https://imagej.nih.gov/ij/, accessed on 5 May 2025).

### 2.6. Metabolic Analysis

The mice underwent a 5 h fasting period before blood collections were performed 3 days prior to transplantation, 10 days after APAP selection, and 5 weeks into the Western diet. The total cholesterol, LDL-C, and triglycerides (TG) were measured in plasma using Pointe Scientific Liquid Reagents kits (Lincoln Park, MI, USA) according to the manufacturer’s protocols. At the endpoint, blood was collected by cardiac or abdominal aorta puncture. The serum was separated and analyzed for metabolic biochemical markers using the 63,772 Custom Chemistry Panel (IDEXX BioAnalytics, Westbrook, MA, USA).

### 2.7. Quantification of Atherosclerosis

Tissues were harvested via whole-body perfusion, postfixed in 10% neutral formalin, and stored in PBS. Aortic root samples were equilibrated in 30% sucrose in PBS, embedded in an Optimal Cutting Temperature compound (Sakura Finetek USA, Inc., Torrance, CA, USA), and frozen in liquid nitrogen vapor. 10-μm thick cryosections were prepared using a cryostat and mounted on positively charged slides. Atherosclerosis was quantified at the level of the aortic sinus per the recommended protocol [33]. Briefly, 64 consecutive 10-μm sections were mounted as an array on eight slides. Each slide contained eight sections starting at the first appearance of the aortic valve and spaced by 80 microns. Slides were stained with Oil Red O stain. The Oil Red O-positive and total plaque areas were measured in at least four sections centered around the left coronary sinus using ImageJ software.

### 2.8. Immunoblot

Liver lysates were prepared by homogenizing 20 mg of liver tissue in ice-cold radioimmunoprecipitation assay buffer (R0278, Sigma Aldrich, St. Louis, MO, USA) supplemented with 1X Halt Protease and Phosphatase Inhibitor Cocktail (78440, Thermo Fisher Scientific, Waltham, MA, USA). Lysates were clarified by centrifugation and then further purified using Zeba Spin Desalting Columns (89882, Thermo Fisher Scientific, Waltham, MA, USA) according to the manufacturer’s instructions. The lysate protein concentrations were quantified using the Pierce BCA Protein Assay (23225, Thermo Fisher Scientific, Waltham, MA, USA) according to the manufacturer’s instructions. Liver lysates containing 20 mg protein were diluted in 2X Laemmli sample buffer (1610737, Bio-Rad, Hercules, CA, USA) supplemented with 5% beta-mercaptoethanol and heated to 95 °C for 10 min, and resolved by SDS-PAGE using 7.5% Criterion^TM^ TGX^TM^ Precast Midi Protein Gels (5671023, Bio-Rad, Hercules, CA, USA) using 1X Tris/Glycine/SDS running buffer (1610732, Bio-Rad, Hercules, CA, USA). Gels were transferred to Immun-Blot PVDF membranes (1620175, Bio-Rad, Hercules, CA, USA) for 16 h at 4 °C in 1X Tris/Glycine buffer (1610734, Bio-Rad, Hercules, CA, USA) with 10% methanol. Membranes were stained with Ponceau S Stain (A40000279, Fisher Scientific, Waltham, MA, USA) for 10 min to confirm transfer, followed by three 1 min washes with distilled water. Membranes were blocked in EveryBlot Blocking Buffer (12010020, Bio-Rad, Hercules, CA, USA) for 30 min at room temperature with gentle agitation and incubated overnight at 4 °C with primary antibodies: mouse anti-mouse heat shock protein-90 (HSP90) (610419, BD Biosciences, San Jose, CA, USA) and rabbit anti-mouse Cytochrome P450 2E1 (ab28146, Abcam, Waltham, MA, USA) diluted in EveryBlot blocking buffer according to manufacturers’ specifications. After washing steps using TBST, membranes were incubated with secondary antibodies: goat anti-mouse IgG-HRP conjugate (AP181P, Sigma, St. Louis, MO, USA) and goat anti-rabbit IgG-HRP conjugate (12-348, Sigma, St. Louis, MO, USA) were diluted in blocking buffer. Protein detection was performed using SuperSignal West Femto chemiluminescent substrate (34096, Thermo Fisher Scientific, Waltham, MA, USA) according to the manufacturer’s instructions. Membrane imaging was conducted using a GelStudio PLUS imaging system (Analytik Jena, Upland, CA, USA). Densitometric analysis was performed using ImageJ software.

### 2.9. Real-Time Quantitative PCR (RT-PCR) Analysis

RNA was isolated from homogenized liver tissue using the TRIzol^TM^ Reagent (15596026, Thermo Fisher Scientific, Waltham, MA, USA) and purified using the Direct-zol RNA Miniprep Plus Kit (R2071, Zymo Research, Irvine, CA, USA). cDNA was generated using the High-Capacity cDNA Reverse Transcription Kit (4368813, Thermo Fisher Scientific, Waltham, MA, USA) and 2 µg of RNA. The transcript levels of each cytochrome P450 enzyme; CYP 3A11, CYP 2E1, and each variant of the CYP 2C subfamily were measured using the Roche FastStart SYBR Green Master Kit (4673484001, Sigma-Aldrich, Burlington, MA, USA) and 250 ng of cDNA with β-Actin as the reference gene. RT-PCR was conducted on the Bio-Rad CFX96^TM^ system using previously established protocols and gene-specific primers as described in [34]. Fold change and relative expression of all genes were calculated using the ΔΔCq method [35].

### 2.10. Statistical Analysis

All statistical analyses were performed using GraphPad Prism software version 10.4.2. Statistical significance was set at *p* < 0.05. Experimental differences in the RT-PCR data were analyzed using two-way ANOVA. For all other experiments, differences between multiple groups were compared using one-way ANOVA, followed by Tukey’s correction for multiple comparisons. For all statistical analyses, * *p* < 0.05, ** *p* < 0.01, *** *p* < 0.001, and **** *p* < 0.0001.

## 3. Results

### 3.1. APAP-Mediated Selection of Electroporated Hepatocytes in C57BL/6J Mice

We previously designed and validated the *Cypor*-aiming guide RNA in mouse hepatocytes [24,25]. First, we validated the selection of Cypor-deficient hepatocytes using APAP administration in wild-type C57BL/6J mice. Plasmids encoding *Cypor*-aiming CRISPR-Cas9 were hydrodynamically injected into mice, then two weeks post-injection, half the mice were subjected to biweekly APAP administration for 8 weeks. Selection was assessed by monitoring ALT levels, measured 6 h after each APAP injection (Supplementary Figure S1A). APAP dose began at 200 mg/kg and increased 10–25 mg/kg each dose until a spike of >1000 U/L was reached. ALT levels were elevated at the start of APAP treatment and spiked with an APAP dose of 275–300 mg/kg. ALT levels returned to baseline with subsequent doses, indicating the expansion of Cypor-deficient hepatocytes in the liver. At 10 days post-APAP treatment, mice were sacrificed, the livers dissected, and the blood collected. IF staining against Cypor protein in liver tissue sections verified selection with the presence of regions consisting of Cypor-deficient hepatocytes (Supplementary Figure S1B). These results show that *Cypor* knockout in hepatocytes, followed by APAP treatment, is a feasible and efficient method for expanding gene-edited hepatocytes in the liver.

We evaluated the extent to which hepatocytes electroporated with *Cypor*-aiming Cas9 RNP are capable of engraftment and in vivo selection using APAP treatment. Hepatocytes were isolated from wild-type C57BL/6J mice, electroporated, and then transplanted into C57BL/6J recipient mice via intrasplenic injection. We observed a high on-target gene editing efficiency of 65% in the electroporated hepatocytes that were plated (Supplementary Figure S1C). After 8 weeks of APAP injections followed by a 10-day recovery period, the mice were sacrificed. The stained liver sections confirmed expansion of Cypor-deficient hepatocytes (Supplementary Figure S1D). To further assess the effects of APAP selection, liver biochemical markers in the serum were analyzed (Supplementary Figure S1E–H) and showed no significant difference between APAP-treated and untransplanted controls for ALT, aspartate aminotransferase (AST), alkaline phosphatase (ALP), total bilirubin (TBIL), and albumin (ALB) levels, indicating that APAP selection did not cause liver damage in transplanted recipients. These findings demonstrate the feasibility of using electroporation ex vivo combined with hepatocyte transplantation and transient APAP treatment to selectively expand gene-edited hepatocytes engrafted in the liver.

### 3.2. Cypor-Deficient Hepatocytes Selected in Ldlr^−/−^ Mice Using APAP Treatment

First, we evaluated the impacts of electroporation on LDL-C uptake using an in vitro assay. Hepatocytes isolated from wild-type C57BL/6J mice were electroporated with *Cypor*-Cas9 RNPs and incubated with pHrodoRed-labeled LDL-C conjugate, and then analyzed using flow cytometry to assess LDL-C uptake (Supplementary Figure S2A). The Cypor-deficient C57BL/6J hepatocytes had a similar uptake in pHrodoRed-LDL-C compared to the untreated wild-type cells. Hepatocytes from *Ldlr*^−/−^ mice showed significantly lower pHrodoRed-LDL-C uptake (Supplementary Figure S2B) than wild-type Cypor-deficient hepatocytes (mean 72% and 115%, respectively, *p* = 0.0228), demonstrating functional impairment of LDL uptake consistent with LDL deficiency. The results further validate that the isolated electroporated hepatocytes are viable and can uptake LDL cholesterol.

**Figure 1:**
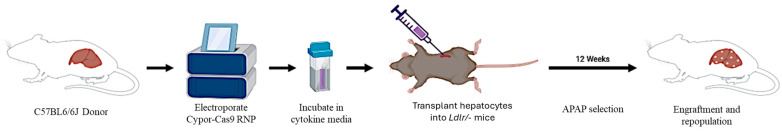
Schematic of experimental setup used in transplant studies. Primary hepatocytes isolated from wild-type mice were electroporated to introduce *Cypor*-CRISPR-Cas9 RNPs and transplanted into *Ldlr^−/−^* recipient mice. After transplantation, recipient mice were treated with APAP to activate selection of Cypor-deficient hepatocytes in vivo.

**Figure 2:**
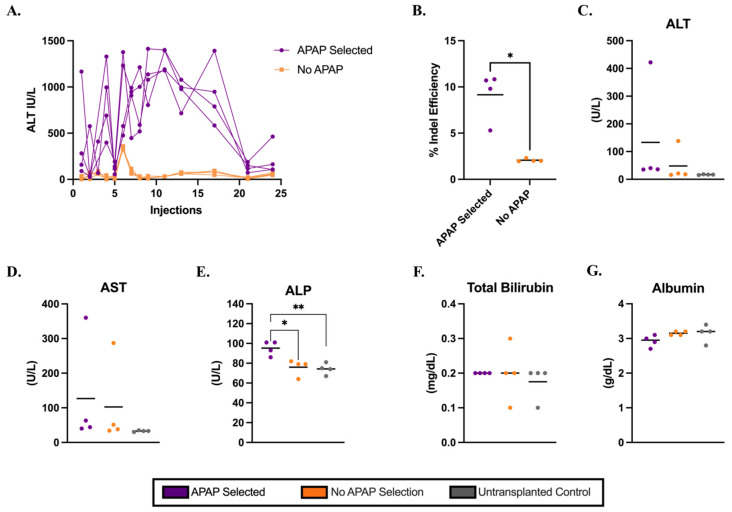
Electroporated hepatocytes isolated from wild-type donor mice engraft in *Ldlr^−/−^* mice with transient APAP administration. (**A**) ALT levels in mice 6 h after APAP injection for mice transplanted with gene-edited Cypor-deficient hepatocytes (*n* = 4). (**B**) Percent of on-target gene editing efficiency measured by indel analysis on gDNA isolated from whole liver homogenates (*n* = 4). Levels of liver biomarkers in serum: (**C**) ALT, (**D**) AST, (**E**) ALP, (**F**) TBIL, and (**G**) albumin, respectively, for APAP-selected mice, no-APAP, and untransplanted controls (*n* = 4). Bars represent the mean. Differences are not significant unless indicated. Levels of significance * *p* < 0.05, and ** *p* < 0.01 (one-way ANOVA with Tukey’s multiple comparison).

Next, we assessed whether hepatocytes isolated from healthy C57BL/6J mice retained their potential to function and clonally expand after electroporation in an *Ldlr*^−/−^ mouse model of FH. *Cypor*-targeting RNPs were delivered into wild-type hepatocytes via electroporation (Supplementary Figure S3) and transplanted into *Ldlr*^−/−^ mice via intrasplenic injection (Figure 1). As a positive control for engraftment, gene-edited hepatocytes were transplanted into *Fah*^−/−^ mice and subjected to NTBC withdrawal to activate repopulation of the liver by transplanted hepatocytes. IHC staining in liver sections from *Fah*^−/−^ recipient mice showed 18% Fah-positive hepatocytes in the liver by 40 days post-transplantation in a system not requiring APAP selection (Supplementary Figure S4). At 12 days post-transplantation, *Ldlr*^−/−^ mice were randomly assigned to receive biweekly APAP injections (Supplementary Table S1). In contrast to C57BL/6J mice, *Ldlr*^−/−^ mice required longer APAP administration to regain baseline ALT levels. The plasma ALT levels remained elevated until 21 doses of APAP were injected, thereafter reducing to baseline until the end of the 12-week selection period (Figure 2A). Mice were sacrificed 10 days after the final injection and the livers were harvested for engraftment analysis. Genomic DNA isolated from homogenized liver tissue of *Ldlr*^−^/^−^ mice was used to quantify on-target gene editing indels, serving as an estimate of liver engraftment. We observed significantly higher engraftment (Figure 2B) based on the indels in APAP-selected mice than in no-APAP transplanted control mice (mean 9% and 2%, respectively, *p* = 0.0121). The engraftment was confirmed by IF staining in liver sections (Supplementary Figure S5). It is important to note that the liver has a zonal dependence for Cypor expression [36], such that Cypor-deficient regions were visible in the no-APAP controls. Consistent with the findings in C57BL/6J mice, the results indicate that electroporated hepatocytes with *Cypor* disrupted are capable of engraftment and selective expansion in vivo in *Ldlr*^−/−^ mice using APAP administration.

### 3.3. Metabolic Biomarkers and Histology Analysis in Ldlr^−/−^ Mice on a Regular Diet

We investigated the impacts of Cypor-deficient hepatocytes selected using 12 weeks of APAP injections in *Ldlr*^−/−^ recipient mice. The biochemical liver panel (Figure 2C–G) analysis revealed no significant differences in biomarker serum levels, except for ALP (Figure 2E). The ALP levels were significantly higher in the APAP-selected mice compared to the no-APAP and untransplanted controls. However, the mean ALP level in APAP-selected mice was 95.2 U/L, which is within the healthy range for mice [37], suggesting no liver damage. The livers harvested from APAP-treated mice displayed a granular appearance (Supplementary Figure S6). Further analysis of the liver pathology using H&E staining in liver tissue sections in APAP-treated mice showed regions with variable amounts of ballooned hepatocytes and inflammatory cells (Supplementary Figure S7, Supplementary Table S2), which were absent in liver sections from no-APAP and untransplanted control mice. The plasma lipid levels were quantified to assess the impact of the APAP selection of Cypor-deficient gene-edited hepatocytes on cholesterol levels in *Ldlr*^−/−^ mice on a regular chow diet. *Ldlr*^−/−^ mice treated with APAP showed 19% lower total cholesterol levels and 9% lower triglyceride levels compared to no-APAP mice (Supplementary Figure S8), and significantly lower total cholesterol levels than untransplanted controls (mean 178 mg/dL and 261 mg/dL, respectively, *p* = 0.0394. Together, these findings indicate that although biochemical markers did not reveal liver damage in mice treated with APAP, the engraftment of Cypor-deficient hepatocytes was associated with liver lipidosis. At the same time, blood lipid levels were reduced in APAP-selected, engraft-recipient mice compared to controls.

### 3.4. Persistence of Gene-Edited Hepatocytes in the Liver After Western Diet

**Figure 3:**
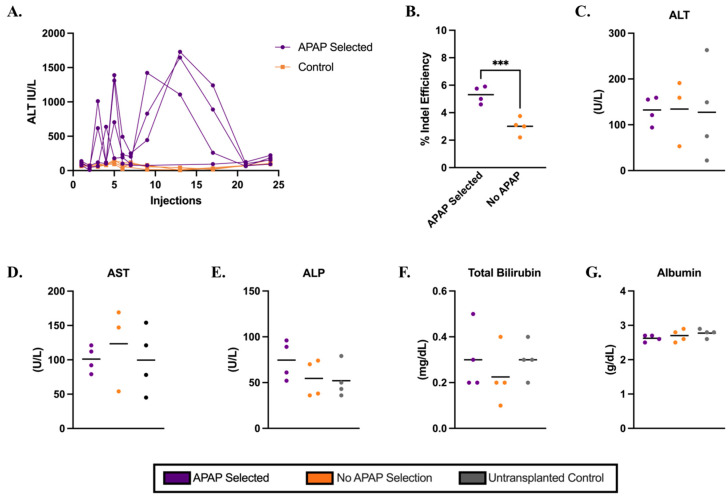
Electroporated hepatocytes isolated from wild-type donor mice engrafted in *Ldlr^−/−^* mice with APAP administration followed by 12-week Western diet. (**A**) ALT levels in mice 6 h after APAP injection for transplanted mice (*n* = 4). (**B**) Percent gene editing efficiency measured by indel analysis in whole liver homogenates (*n* = 4). Levels of liver biomarkers in serum: (**C**) ALT, (**D**) AST, (**E**) ALP, (**F**) TBIL, and (**G**) albumin, respectively, for APAP-selected, no-APAP, and untransplanted controls (*n* = 4). Bars represent the mean. Differences are not significant unless indicated. Levels of significance *** *p* < 0.001 (one-way ANOVA with Tukey’s multiple comparison).

To further evaluate the potential therapeutic effect of this approach, we repeated the transplantation of Cypor-deficient wild-type hepatocytes into *Ldlr*^−/−^ mice. At 12 days post-transplantation, mice were randomly assigned to undergo APAP selection (Supplementary Table S1) and subsequently placed on a Western diet to induce atherosclerosis. Transplanted electroporated hepatocytes were plateable and had a mean gene editing efficiency of 95% and high viability (Supplementary Figure S9). As an additional indicator of high cell quality, electroporated hepatocytes transplanted into *Fah*^−/−^ mice underwent one cycle of NTBC treatment, were maintained off NTBC for 25 days, and showed 25% engraftment in the liver estimated by the on-target indels in the liver (Supplementary Figure S10). In *Ldlr*^−/−^ mice, ALT levels were monitored as an indicator of selection. After an initial spike, ALT levels rapidly returned to baseline after 21 APAP injections (Figure 3A). At 10 days after the final APAP dose, mice were switched to a Western diet and maintained on it for 12 weeks for aortic atherosclerosis plaque formation. Indel analysis in gDNA extracted from the liver homogenates (Figure 3B) revealed slightly higher indels in APAP-selected mice than the no-APAP controls (mean 5% and 3%, respectively, *p* = 0.0005); however, the IF staining in liver sections (Supplementary Figure S11) showed no obvious differences between the APAP-treated mice and no-APAP controls. Together, these results indicate lower engraftment in mice maintained on the Western diet compared to those sacrificed following APAP selection, suggesting a loss of gene-edited, Cypor-deficient hepatocytes by the end of the 12-week Western diet.

### 3.5. Metabolic Markers and Atherosclerosis in Ldlr^−/−^ Recipient Mice on a Western Diet

**Figure 4:**
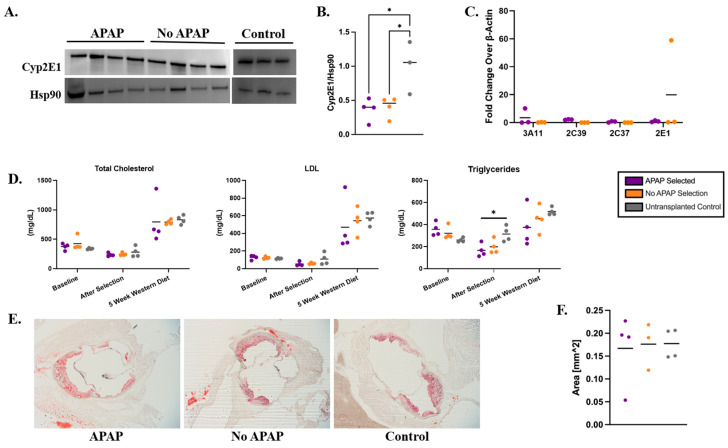
Therapeutic effects and impact on drug metabolism by APAP selection of Cypor-deficient hepatocytes in *Ldlr^−/−^* mice on a Western diet. (**A**) Immunoblots used to determine (**B**) expression of CypP2E1 relative to Hsp90 in APAP-treated, no-APAP, and untransplanted control mice measured with densitometry analysis (*n* = 4). (**C**) Relative expression levels (2^−ΔΔCq^) of four cytochrome P450 enzymes (*n* = 3). (**D**) Total cholesterol, LDL-C, and triglyceride levels in blood plasma at different timepoints throughout the study (*n* = 4). (**E**) Representative images of aortic plaque stained with Oil Red O used to measure (**F**) the area of aortic plaques (*n* = 4). Bars represent the mean. Differences are not significant unless indicated. Levels of significance * *p* < 0.05 (one-way ANOVA with Tukey’s multiple comparison).

There were no statistically significant differences in ALT, AST, ALP, TBIL, or ALB levels between APAP-selected and control groups, suggesting that the 12-week APAP regimen combined with a 12-week Western diet did not induce liver toxicity in *Ldlr*^−/−^ mice as assessed by serum biochemical markers (Figure 3C–G). However, the livers harvested from APAP-selected mice were fragile and exhibited a granular appearance (Supplementary Figure S12), indicating pathology. Analysis of H&E-stained liver sections confirmed that the 12-week Western diet induced steatosis (Supplementary Figure S13), although the mice treated with APAP showed massive, generalized steatosis, whereas no-APAP controls showed more micro-vesicular steatosis (Supplementary Table S3). Analysis of additional biochemical markers in the serum revealed no significant differences between experimental mice and controls, indicating that APAP treatment did not result in detectable damage to liver or kidney function (Supplementary Figure S14). To further evaluate whether Cypor deficiency was contributing to compensatory effects in Cyp enzymes, we measured Cyp2E1 protein levels, a key enzyme in APAP metabolism, in liver lysates using immunoblot (Figure 4A,B) and the mRNA levels of Cyp3E11, Cyp2E1, Cyp2C37, and Cyp2C39 using RT-PCR (Figure 4C). The original immunoblot images can be found in Supplementary Figure S15. We observed no difference in the liver Cyp enzyme levels between the APAP-selected mice and no-APAP transplanted controls (Figure 4B,C), indicating that the overall Cyp expression was likely not impacted by Cypor knockdown combined with APAP administration.

To investigate the effects of the Cypor-deficient hepatocytes on blood cholesterol levels in *Ldlr*^−/−^ mice treated with APAP, blood was collected at the following time points: 3 days prior to transplantation, 10 days after the final APAP dose, and 5 weeks on the Western diet. Plasma was separated from the blood and analyzed for total cholesterol, LDL-C, and triglyceride levels. No significant differences were observed in baseline levels, validating the consistency of the lipid tests (Figure 4D). A significant decrease in triglyceride levels was observed in APAP-selected mice compared to untransplanted controls after selection (mean 166 mg/dL and 314 mg/dL, respectively, *p* = 0.0242). At 5 weeks on the Western diet, LDL-C and triglyceride levels were 18% and 52% lower than untransplanted mice, respectively. To ensure the development of atherosclerosis [38], mice were maintained on a Western diet for an additional 7 weeks and the aortic root was dissected and stained to quantify plaque formation (Figure 4E). We observed no difference in the plaque areas between the APAP-selected mice, no-APAP, and untransplanted control groups (Figure 4F). Together, our results indicate that APAP selection of Cypor-deficient hepatocytes reduces triglyceride and LDL-C levels in mice maintained on a Western diet for 5 weeks. However, due to lipid accumulation and associated lipotoxicity, Cypor-deficient hepatocytes are unable to efficiently clear lipids, leading to massive steatosis, loss of edited hepatocytes, and diminished protection against the development of atherosclerosis.

## 4. Discussion

Based on these findings, we confirmed that hepatocytes isolated from C57BL/6J mice and electroporated with *Cypor*-targeting Cas9 RNPs can successfully engraft and repopulate the liver in C57BL/6J and *Ldlr*^−/−^recipient mice following transient APAP administration to levels predicted to provide therapeutic benefit. As confirmed by liver biomarker analysis, we observed no evidence of APAP-induced liver toxicity in C57BL/6J mice. In contrast, *Ldlr*^−/−^ mice showed evidence of liver pathology. The recipient *Ldlr*^−/−^ mice treated with APAP showed significantly higher ALP levels compared to controls at 10 days post-APAP treatment, while other liver biochemistry markers showed no differences between the APAP-treated mice and controls (Figure 2). However, the mean ALP level remained within the normal physiological range for mice, indicating no change in liver function. With a longer recovery period following APAP selection, the elevated ALP would likely have returned to the same level as the controls. However, histological analysis of liver sections revealed the abnormal formation of lipid-filled nodules in APAP-selected mice on a regular chow diet, which is indicative of lipidosis in *Ldlr*^−/−^recipient mice due to Cypor deficiency.

Our results indicate that the engraftment of Cypor-deficient healthy hepatocytes expressing Ldlr in the liver of *Ldlr*^−/−^ recipient mice slightly lowers lipid levels. After selection, we observed a reduction in the total cholesterol (16%), LDL-C (51%), and triglycerides (47%) in the recipients selected with APAP compared to the untransplanted controls on a regular chow diet (Figure 4). The reduced lipid levels persisted when *Ldlr*^−/−^ recipient mice were switched to a Western diet following APAP selection. After 5 weeks on the Western diet, we observed reductions in total cholesterol (5%), LDL-C (18%), and triglyceride levels (52%) compared to the untransplanted controls. However, after 12 weeks on the Western diet, there was a loss of gene-edited hepatocytes (Figure 3) compared to the levels after selection (Figure 2). The decrease in lipid levels was insufficient to protect against atherosclerosis, as indicated by the aortic plaque areas.

On the regular diet, histological examination via H&E staining indicated liver lipidosis and modest inflammation in recipient *Ldlr*^−/−^ mice selected with APAP. However, we observed normal liver morphology in controls on the regular diet. On the Western diet, transplanted mice treated with APAP exhibited more severe steatosis with inflammation than the controls. These findings suggest that the transplanted Cypor-deficient hepatocytes caused the accumulation of lipids in the liver and that the Western diet exacerbates the lipid content. The LDL-C in vitro assay (Supplementary Figure S2) results confirm that the Cypor deficiency in gene-edited hepatocytes results in enhanced LDL-C uptake compared to unedited wild-type hepatocytes. Prior studies in hepatic Cypor-null mice reveal that Cypor deficiency results in elevated lipid content with neutrophilic cell infiltrates, hepatitis in the liver, as well as lower serum cholesterol and triglycerides due to inhibition of de novo cholesterol biosynthesis and bile acid biosynthetic pathways [39,40,41]. Hepatic cytochrome p450 enzymes are involved in cholesterol synthesis, such as Cyp51 [42], and lipid conversion into bile acids in the bile acid synthesis pathways, such as Cyp7A1 [43], which requires the obligate co-factor Cypor for activity. The histology and lipid results in the *Ldlr^−/−^* recipient mice selected with APAP are similar to the hepatic Cypor-null and Cypor-low mouse strains characterized in [39,40,41]. However, unlike studies conducted in mice with liver-specific Cypor knockout, we did not observe any change in cytochrome P450 protein and mRNA expression levels in the liver between APAP-treated and no-APAP controls determined by immunoblotting and RT-PCR (Figure 4). This suggests no compensatory effects from selecting Cypor-deficient hepatocytes in the liver using transient APAP administration. However, while expression levels may remain unchanged, enzyme activity is affected due to the loss of their essential cofactor resulting from the knockout. The accumulation of lipids in the liver suggests that Cypor deficiency in the engrafted hepatocytes impaired cholesterol clearance due to broad inhibition of Cyp enzymes required for bile acid metabolism. Furthermore, the reduced indel frequency observed after the 12-week Western diet, compared to APAP selection, suggests that lipid accumulation in donor *Ldlr*^+/+^ hepatocytes following prolonged exposure to a high cholesterol diet compromised the long-term viability and resulted in the clearance of gene-edited hepatocytes.

The results from our study raise concerns about applying the Cypor disruption APAP selection approach for expanding gene-edited hepatocytes in the liver to treat dyslipidemias. In our prior study, APAP selection of Cypor-deficient healthy hepatocytes resulted in a high level of liver repopulation by healthy hepatocytes to correct the PKU mouse model [24]. The Cypor-deficient hepatocytes expanded to approximately 14% engraftment, restoring blood phenylalanine concentrations to healthy levels. However, in contrast to the *Ldlr*^−/−^ mice, lipid accumulation and steatosis were not observed in the PKU or C57BL/6J recipient mice [24,25], suggesting strain-dependent effects of Cypor deficiency of the engrafted hepatocytes. In PKU mice, cholesterol metabolism can be compensated in native Cypor-expressing hepatocytes in the liver, but this is not the case in the *Ldlr*^−/−^ mice. The accumulated lipids in Cypor-deficient hepatocytes indicate that the engrafted Ldlr-expressing hepatocytes took up excess LDL-C but could not clear the lipids due to the impaired cholesterol conversion of lipids into bile acids, leading to lipotoxicity. Therefore, the approach involving transplantation of Cypor-deficient healthy hepatocytes is feasible for PKU, but unsuitable for treating HoFH, indicating that a one-size-fits-all gene editing strategy is not viable for inherited metabolic liver diseases. The consequences of gene editing approaches that involve metabolic reprogramming of hepatocytes must be carefully considered in the context of the targeted disease because some level of toxicity in the native hepatocytes is required for the donor hepatocytes to expand in the liver. The liver injury must be sufficient to create space for the donor hepatocytes to grow, but not so severe that it causes liver failure [17]. An alternative approach for HoFH would involve targeting specific Cyp enzymes involved in APAP metabolism, rather than Cypor, which would confer resistance to transient APAP treatment while avoiding the detrimental effects on lipid metabolism and cholesterol clearance [44,45]. Our findings suggest that transient administration of APAP may also confer a selective advantage for the expansion of hepatocytes deficient in APAP-metabolizing CYP enzymes in *Ldlr*^−/−^ recipients, which is a hypothesis we will explore in follow-up studies. Moreover, the findings of this study highlight the utility of electroporation in hepatocytes as a platform for evaluating cell-based gene editing strategies aimed at treating inherited metabolic liver disorders.

## 5. Conclusions

We previously demonstrated that *Cypor*-aiming CRISPR–Cas9-mediated gene editing in healthy hepatocytes using nonviral delivery, coupled with transient APAP administration for the expansion of gene-edited hepatocytes in the liver, could cure a mouse model of PKU. In this study, we investigate electroporation-mediated delivery of Cas9 RNPs into hepatocytes as a cell therapy approach, combined with APAP-mediated selection, in a mouse model of homozygous familial hypercholesterolemia (*Ldlr*^−/−^). The results show that while CRISPR–Cas9-mediated disruption of *Cypor* conferred APAP resistance and enabled the robust engraftment of gene-edited hepatocytes, achieving up to 13% liver engraftment, and reduced lipid levels, Cypor deficiency results in the accumulation of lipids in the liver and exacerbated steatosis in *Ldlr*^−/−^ mice on a Western diet. Further, the engrafted Ldlr-expressing hepatocytes did not mitigate aortic atherosclerosis in *Ldlr*^−/−^ mice. The effects of Cypor deficiency on lipid metabolism and cholesterol clearance could not be compensated by unedited hepatocytes in the livers of *Ldlr*^−/−^ mice, indicating that while this approach was effective in the PKU mouse model, transplantation of Cypor-deficient hepatocytes is not suitable for treating HoFH. These findings suggest that a one-size-fits-all gene editing strategy may not be universally applicable for treating inherited metabolic liver disorders. Moving forward, tailored gene editing and selection strategies may be needed for different liver disorders.

## Data Availability

Data is contained within the article or Supplementary Materials.

## Supplementary Materials

The following supporting information can be downloaded at: https://www.mdpi.com/article/10.3390/biology14081091/s1, Figure S1: Preliminary results of APAP selection in C57BL/6J recipient mice; Figure S2: Cypor-deficient hepatocytes show increased uptake compared to *Ldlr*^−/−^ hepatocytes; Figure S3: In vitro data from plated C57BL/6J donor hepatocytes used in APAP selection study; Figure S4: Cypor-deficient hepatocytes transplanted into *Fah*^−/−^ mice as engraftment controls for the APAP selection study; Figure S5: Representative Cypor-IF stained liver tissue for *Ldlr*^−/−^ recipient mice in the APAP selection study; Figure S6: Gross liver harvested from *Ldlr*^−/−^ recipient mice in the APAP selection study; Figure S7: Representative H&E-stained liver histology for *Ldlr*^−/−^ recipient mice in the APAP selection study; Figure S8: Lipid levels in blood plasma after 12 weeks of APAP selection; Figure S9: In vitro data from plated C57BL/6J donor hepatocytes used in APAP selection study with western diet; Figure S10: Selection of Cypor-deficient hepatocytes in *Fah*^−/−^ mice via NTBC cycling as engraftment controls for APAP selection study with western diet; Figure S11: Representative Cypor-IF stained liver histology for *Ldlr*^−/−^ recipient mice in the APAP Western Diet selection study; Figure S12: Gross liver images of *Ldlr*^−/−^ mice in APAP selection study with western diet; Figure S13: Representative H&E-stained liver histology for *Ldlr*^−/−^ recipient mice in the APAP selection study with western diet; Figure S14: Liver and kidney panels for *Ldlr*^−/−^ mice subjected to APAP selection and placed on a western diet; Figure S15: Uncropped original immunoblot images corresponding to the data shown in main Figure 4A; Table S1: List of mouse IDs and respective experimental condition and APAP dosage maintained throughout study; Table S2: Histological assessment of H&E-stained histology images of the liver for APAP selection study; Table S3: Histological assessment of H&E-stained histology images of the liver for APAP selection study with western diet.

## Abbreviations

The following abbreviations are used in this manuscript:

ALB	Albumin
ALP	Alkaline phosphatase
ALT	Alanine aminotransferase
APAP	Acetaminophen
AST	Aspartate aminotransferase
Cypor	Cytochrome P450 oxidoreductase
Fah	Fumarylacetoacetate hydrolase
FH	Familial hypercholesterolemia
H&E	Hematoxylin and eosin
HoFH	Homozygous familial hypercholesterolemia
IF	Immunofluorescent
IHC	Immunohistochemistry
IP	Intraperitoneal
LDL-C	Low density lipoprotein—cholesterol
Ldlr	Low density lipoprotein receptor
NTBC	2-nitro-4-trifluoromethylbenzoyl-1,3-cyclohexanedione
RNP	Ribonucleoprotein
RT-PCR	Real-time quantitative PCR
TBIL	Total bilirubin
TG	Triglycerides
WT	Wild-type

## References

[1] Cuchel M., Bruckert E., Ginsberg H.N., Raal F.J., Santos R.D., Hegele R.A., Kuivenhoven J.A., Nordestgaard B.G., Descamps O.S., Steinhagen-Thiessen E. (2014). Homozygous familial hypercholesterolaemia: New insights and guidance for clinicians to improve detection and clinical management. A position paper from the Consensus Panel on Familial Hypercholesterolaemia of the European Atherosclerosis Society. Eur. Heart J..

[2] Chaudhry A., Trinder M., Vesely K., Cermakova L., Jackson L., Wang J., Hegele R.A., Brunham L.R. (2023). Genetic Identification of Homozygous Familial Hypercholesterolemia by Long-Read Sequencing Among Patients With Clinically Diagnosed Heterozygous Familial Hypercholesterolemia. Circ. Genom. Precis. Med..

[3] Bajaj A., Cuchel M. (2022). Advancements in the Treatment of Homozygous Familial Hypercholesterolemia. J. Atheroscler. Thromb..

[4] Vishwanath R., Hemphill L.C. (2014). Familial hypercholesterolemia and estimation of US patients eligible for low-density lipoprotein apheresis after maximally tolerated lipid-lowering therapy. J. Clin. Lipidol..

[5] Risk of fatal coronary heart disease in familial hypercholesterolaemia (1991). Scientific Steering Committee on behalf of the Simon Broome Register Group. BMJ.

[6] Hemphill L., Goldberg A., Hovingh K., Cohen J., Karalis D.G. (2020). Recognition and Treatment of Homozygous Familial Hypercholesterolemia by Primary Care Physicians: A Survey from the National Lipid Association. J. Gen. Intern. Med..

[7] Kayikcioglu M., Tokgozoglu L. (2022). Current Treatment Options in Homozygous Familial Hypercholesterolemia. Pharmaceuticals.

[8] Santos R.D., Gidding S.S., Hegele R.A., Cuchel M.A., Barter P.J., Watts G.F., Baum S.J., Catapano A.L., Chapman M.J., Defesche J.C. (2016). Defining severe familial hypercholesterolaemia and the implications for clinical management: A consensus statement from the International Atherosclerosis Society Severe Familial Hypercholesterolemia Panel. Lancet Diabetes Endocrinol..

[9] Raal F.J., Pilcher G.J., Panz V.R., van Deventer H.E., Brice B.C., Blom D.J., Marais A.D. (2011). Reduction in mortality in subjects with homozygous familial hypercholesterolemia associated with advances in lipid-lowering therapy. Circulation.

[10] Kroon A.A., van’t Hof M.A., Demacker P.N., Stalenhoef A.F. (2000). The rebound of lipoproteins after LDL-apheresis. Kinetics and estimation of mean lipoprotein levels. Atherosclerosis.

[11] Serrano M.T., Sabroso S., Esteban L.M., Berenguer M., Fondevila C., Lorente S., Cortés L., Sanchez-Antolin G., Nuño J., De la Rosa G. (2022). Mortality and Causes of Death After Liver Transplantation: Analysis of Sex Differences in a Large Nationwide Cohort. Transpl. Int..

[12] Maiorana A., Nobili V., Calandra S., Francalanci P., Bernabei S., El Hachem M., Monti L., Gennari F., Torre G., de Ville de Goyet J. (2011). Preemptive liver transplantation in a child with familial hypercholesterolemia. Pediatr. Transplant..

[13] Arnon R., Kerkar N., Davis M.K., Anand R., Yin W., Gonzalez-Peralta R.P., for the SPLIT Research Group (2010). Liver transplantation in children with metabolic diseases: The studies of pediatric liver transplantation experience. Pediatr. Transplant..

[14] Luo Y., Hou Y., Zhao W., Yang B. (2024). Recent progress in gene therapy for familial hypercholesterolemia treatment. iScience.

[15] Grossman M., Rader D.J., Muller D.W.M., Kolansky D.M., Kozarsky K., Clark B.J., Stein E.A., Lupien P.J., Brewer H.B., Raper S.E. (1995). A pilot study of ex vivo gene therapy for homozygous familial hypercholesterolaemia. Nat. Med..

[16] Williams R.S. (1995). Human gene therapy—Of tortises and hares. Nat. Med..

[17] Vonada A., Grompe M. (2024). In vivo selection of hepatocytes. Hepatology.

[18] Sun Z., Yuan X., Wu J., Wang C., Zhang K., Zhang L., Hui L. (2023). Hepatocyte transplantation: The progress and the challenges. Hepatol. Commun..

[19] Gerriets V., Anderson J., Patel P., Nappe T.M. (2025). Acetaminophen. StatPearls.

[20] Mazaleuskaya L.L., Sangkuhl K., Thorn C.F., FitzGerald G.A., Altman R.B., Klein T.E. (2015). PharmGKB summary: Pathways of acetaminophen metabolism at the therapeutic versus toxic doses. Pharmacogenet. Genom..

[21] Xia C., Panda S.P., Marohnic C.C., Martasek P., Masters B.S., Kim J.J. (2011). Structural basis for human NADPH-cytochrome P450 oxidoreductase deficiency. Proc. Natl. Acad. Sci. USA.

[22] Agrawal S., Murray B.P., Khazaeni B. (2025). Acetaminophen Toxicity. StatPearls.

[23] Barahman M., Asp P., Roy-Chowdhury N., Kinkhabwala M., Roy-Chowdhury J., Kabarriti R., Guha C. (2019). Hepatocyte Transplantation: Quo Vadis?. Int. J. Radiat. Oncol. Biol. Phys..

[24] Vonada A., Wakefield L., Martinez M., Harding C.O., Grompe M., Tiyaboonchai A. (2024). Complete correction of murine phenylketonuria by selection-enhanced hepatocyte transplantation. Hepatology.

[25] Vonada A., Tiyaboonchai A., Nygaard S., Posey J., Peters A.M., Winn S.R., Cantore A., Naldini L., Harding C.O., Grompe M. (2021). Therapeutic liver repopulation by transient acetaminophen selection of gene-modified hepatocytes. Sci. Transl. Med..

[26] Gibson J., Dhungana A., Pokhrel M., Arthur B., Suresh P., Adebayo O., Cottle R.N. (2025). Validation of Clinical-Grade Electroporation Systems for CRISPR-Cas9-Mediated Gene Therapy in Primary Hepatocytes for the Correction of Inherited Metabolic Liver Disease. Cells.

[27] Rathbone T., Ates I., Fernando L., Addlestone E., Lee C.M., Richards V.P., Cottle R.N. (2022). Electroporation-Mediated Delivery of Cas9 Ribonucleoproteins Results in High Levels of Gene Editing in Primary Hepatocytes. Cris. J..

[28] Ates I., Stuart C., Rathbone T., Barzi M., He G., Major A.M., Shankar V., Lyman R.A., Angner S.S., Mackay T.F.C. (2024). Ex vivo gene editing and cell therapy for hereditary tyrosinemia type 1. Hepatol. Commun..

[29] Rathbone T., Ates I., Stuart C., Parker T., Cottle R.N. (2022). Electroporation-mediated Delivery of Cas9 Ribonucleoproteins and mRNA into Freshly Isolated Primary Mouse Hepatocytes. J. Vis. Exp..

[30] Brinkman E.K., Chen T., Amendola M., van Steensel B. (2014). Easy quantitative assessment of genome editing by sequence trace decomposition. Nucleic Acids Res..

[31] Xu L., Liu Y., Han R. (2019). BEAT: A Python Program to Quantify Base Editing from Sanger Sequencing. Cris. J..

[32] Grompe M. (2006). Principles of therapeutic liver repopulation. J. Inherit. Metab. Dis..

[33] Daugherty A., Tall A.R., Daemen M., Falk E., Fisher E.A., Garcia-Cardena G., Lusis A.J., Owens A.P., Rosenfeld M.E., Virmani R. (2017). Recommendation on Design, Execution, and Reporting of Animal Atherosclerosis Studies: A Scientific Statement From the American Heart Association. Arterioscler. Thromb. Vasc. Biol..

[34] Zhang Q.Y., Dunbar D., Kaminsky L.S. (2003). Characterization of mouse small intestinal cytochrome P450 expression. Drug Metab. Dispos..

[35] Livak K.J., Schmittgen T.D. (2001). Analysis of Relative Gene Expression Data Using Real-Time Quantitative PCR and the 2^−ΔΔCT^ Method. Methods.

[36] Oinonen T., Lindros K.O. (1998). Zonation of hepatic cytochrome P-450 expression and regulation. Biochem. J..

[37] Loeb W.F., Quimbly F.W. (1999). The Clinical Chemistry of Laboratory Animals.

[38] Getz G.S., Reardon C.A. (2016). Do the Apoe^−/−^ and Ldlr^−/−^ Mice Yield the Same Insight on Atherogenesis?. Arterioscler. Thromb. Vasc. Biol..

[39] Gu J., Weng Y., Zhang Q.Y., Cui H., Behr M., Wu L., Yang W., Zhang L., Ding X. (2003). Liver-specific deletion of the NADPH-cytochrome P450 reductase gene: Impact on plasma cholesterol homeostasis and the function and regulation of microsomal cytochrome P450 and heme oxygenase. J. Biol. Chem..

[40] Weng Y., DiRusso C.C., Reilly A.A., Black P.N., Ding X. (2005). Hepatic gene expression changes in mouse models with liver-specific deletion or global suppression of the NADPH-cytochrome P450 reductase gene. Mechanistic implications for the regulation of microsomal cytochrome P450 and the fatty liver phenotype. J. Biol. Chem..

[41] Henderson C.J., Otto D.M., Carrie D., Magnuson M.A., McLaren A.W., Rosewell I., Wolf C.R. (2003). Inactivation of the hepatic cytochrome P450 system by conditional deletion of hepatic cytochrome P450 reductase. J. Biol. Chem..

[42] Stromstedt M., Rozman D., Waterman M.R. (1996). The ubiquitously expressed human CYP51 encodes lanosterol 14 alpha-demethylase, a cytochrome P450 whose expression is regulated by oxysterols. Arch. Biochem. Biophys..

[43] Schwarz M., Russell D.W., Dietschy J.M., Turley S.D. (1998). Marked reduction in bile acid synthesis in cholesterol 7alpha-hydroxylase-deficient mice does not lead to diminished tissue cholesterol turnover or to hypercholesterolemia. J. Lipid Res..

[44] Zaher H., Buters J.T., Ward J.M., Bruno M.K., Lucas A.M., Stern S.T., Cohen S.D., Gonzalez F.J. (1998). Protection against acetaminophen toxicity in CYP1A2 and CYP2E1 double-null mice. Toxicol. Appl. Pharmacol..

[45] Zong H., Armoni M., Harel C., Karnieli E., Pessin J.E. (2012). Cytochrome P-450 CYP2E1 knockout mice are protected against high-fat diet-induced obesity and insulin resistance. Am. J. Physiol. Endocrinol. Metab..

