# Supplementary material for: One Shock, Not One Cure: Electroporation Reveals Disease-Specific Constraints in Hepatocyte Gene Editing Therapy
Source: Biology (Basel). 2025 Aug 20;14(8):1091. doi: 10.3390/biology14081091 (PMC12383315; doi:10.3390/biology14081091)
Supplement: Supplementary file 1 [file biology-14-01091-s001.zip › biology-3716354-supplementary.pdf]

One Shock, Not One Cure: Electroporation Reveals Disease-Specific Constraints in Hepatocyte Gene Editing Therapy

Callie Clark [1], Menam Pokhrel [1], Benjamin Arthur [1], Pramita Suresh [1], Ilayda Ates [1], Justin Gibson [1], Abishek Dhungana [1], Ryan Mehlem [1], Andrew Boysia [1], Mugdha V. Padalkar [2], Achala Pokhrel [3], Jing Echesabal-Chen [3], Anne Vonada [4], Alexis Stamatikos [3], Olga V. Savinova [2], Markus Grompe [4] and Renee N. Cottle [1,5,*]

1 Department of Bioengineering, Clemson University, Clemson, SC 29634, USA; calliedclark@gmail.com (C.C.); mpokhre@clemson.edu (M.P.); bbarthu@clemson.edu (B.A.); pramitasuresh@icloud.com (P.S.); ilayda.ates9@gmail.com (I.A.); jrg6@g.clemson.edu (J.G.); adhunga@clemson.edu (A.D.); rmehlem@clemson.edu (R.M.); aboysia@g.clemson.edu (A.B.)
2 Department of Biomedical Sciences, New York Institute of Technology College of Osteopathic Medicine, Old Westbury, NY 115680, USA; mpadalka@nyit.edu (M.V.P.); osavinov@nyit.edu (O.V.S.)
3 Department of Food, Nutrition, and Packaging Sciences, Clemson University, Clemson, SC 29634, USA; achalap@clemson.edu (A.P.); jchen11@clemson.edu (J.E.-C.); adstama@clemson.edu (A.S.)
4 Papé Family Pediatric Research Institute, Oregon Health & Science University, Portland, OR 97239, USA; vonada@ohsu.edu (A.V.); grompem@ohsu.edu (M.G.)
5 Center for Human Genetics, Clemson University, Greenwood, SC 29646, USA
* Correspondence: rcottle@clemson.edu

Table of Contents

Supplementary Methods

Supplementary Method 1. Hydrodynamic tail vein injection

Supplementary Figures

Supplementary Figure S1. Preliminary results of APAP selection in C57BL/6J recipient mice.

Supplementary Figure S2. Cypor-deficient hepatocytes show increased uptake compared to *Ldlr*$^{-/-}$ hepatocytes.

Supplementary Figure S3. *In vitro* data from plated C57BL/6J donor hepatocytes used in APAP selection study.

Supplementary Figure S4. Cypor-deficient hepatocytes transplanted into *Fah*$^{-/-}$ mice as engraftment controls for the APAP selection study.

Supplementary Figure S5. Representative Cypor-IF stained liver tissue for *Ldlr*$^{-/-}$ recipient mice in the APAP selection study.

Supplementary Figure S6. Gross liver harvested from *Ldlr*$^{-/-}$ recipient mice in the APAP selection study.

Supplementary Figure S7. Representative H&E-stained liver histology for *Ldlr*$^{-/-}$ recipient mice in the APAP selection study.

Supplementary Figure S8. Lipid levels in blood plasma after 12 weeks of APAP selection.

Supplementary Figure S9. *In vitro* data from plated C57BL/6J donor hepatocytes used in APAP selection study with western diet.

Supplementary Figure S10. Selection of Cypor-deficient hepatocytes in *Fah*$^{-/-}$ mice via NTBC cycling as engraftment controls for APAP selection study with western diet.

Supplementary Figure S11. Representative Cypor-IF stained liver histology for *Ldlr*$^{-/-}$ recipient mice in the APAP Western Diet selection study.

Supplementary Figure S12. Gross liver images of *Ldlr*$^{-/-}$ mice in APAP selection study with western diet.

Supplementary Figure S13. Representative H&E-stained liver histology for *Ldlr*$^{-/-}$ recipient mice in the APAP selection study with western diet.

Supplementary Figure S14. Liver and kidney panels for *Ldlr*$^{-/-}$ mice subjected to APAP selection and placed on a western diet.

Supplementary Figure S15. Uncropped original immunoblot images corresponding to the data shown in main Figure 4A.

Supplementary Tables

Supplementary Table S1. List of mouse IDs and respective experimental condition and APAP dosage maintained throughout study.

Supplementary Table S2. Histological assessment of H&E-stained histology images of the liver for APAP selection study.

Supplementary Table S3. Histological assessment of H&E-stained histology images of the liver for APAP selection study with western diet.

Supplementary Methods 1. Hydrodynamic tail vein injection

40 µg of plasmid encoding *Streptococcus pyogenes* Cas9 (spCas9) and a U6 promoter-driven gRNA targeting *Cypor* (pX330-Cypor gRNA) was diluted in PBS to a final volume equivalent to 10% of the recipient mouse's body weight. Heat source was placed on tail of anesthetized mouse for up to 5 minutes. The plasmid solution was administered via hydrodynamic tail vein injection using a 30-guage insulin syringe.

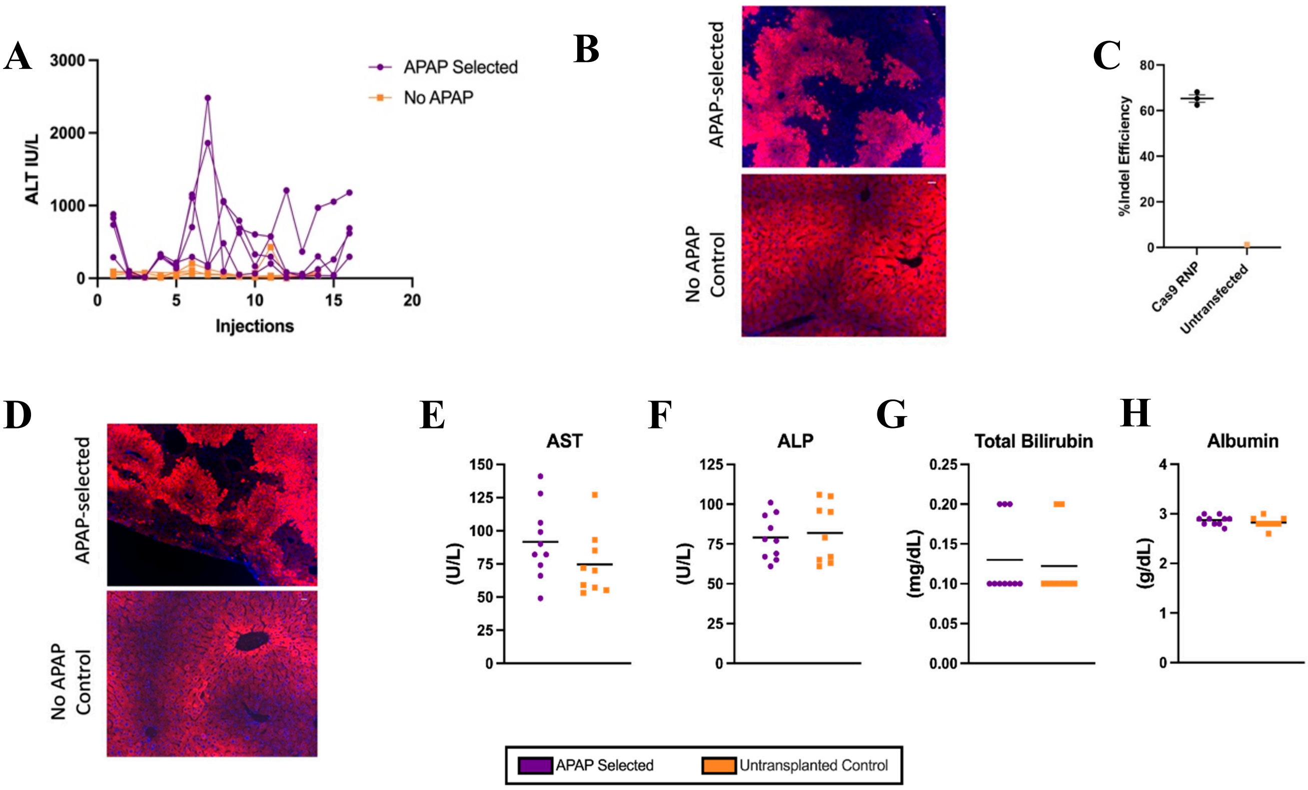

Supplementary Figure S1. Preliminary results of APAP selection in C57BL/6J recipient mice. (A) ALT levels for APAP selected and non-selected mice hydrodynamically injected with Cypor-aiming plasmids. **(B)** IF stained images against Cypor in liver tissue harvested after delivery of Cypor-aiming plasmids and APAP selection. **(C)** *In vitro* on-target indels in electroporated hepatocytes used for transplantation. **(D)** IF stained images against Cypor in liver tissue harvested after transplantation of Cypor-edited hepatocytes and APAP selection. **(E-H)** Liver biomarker analysis from blood collected at sacrifice after transplantation and APAP selection (n = 9). Differences are not significant unless indicated. Levels of significance *P < 0.05, **P < 0.01, ***P < 0.001, ****P < 0.0001 (unpaired Student's t-test).

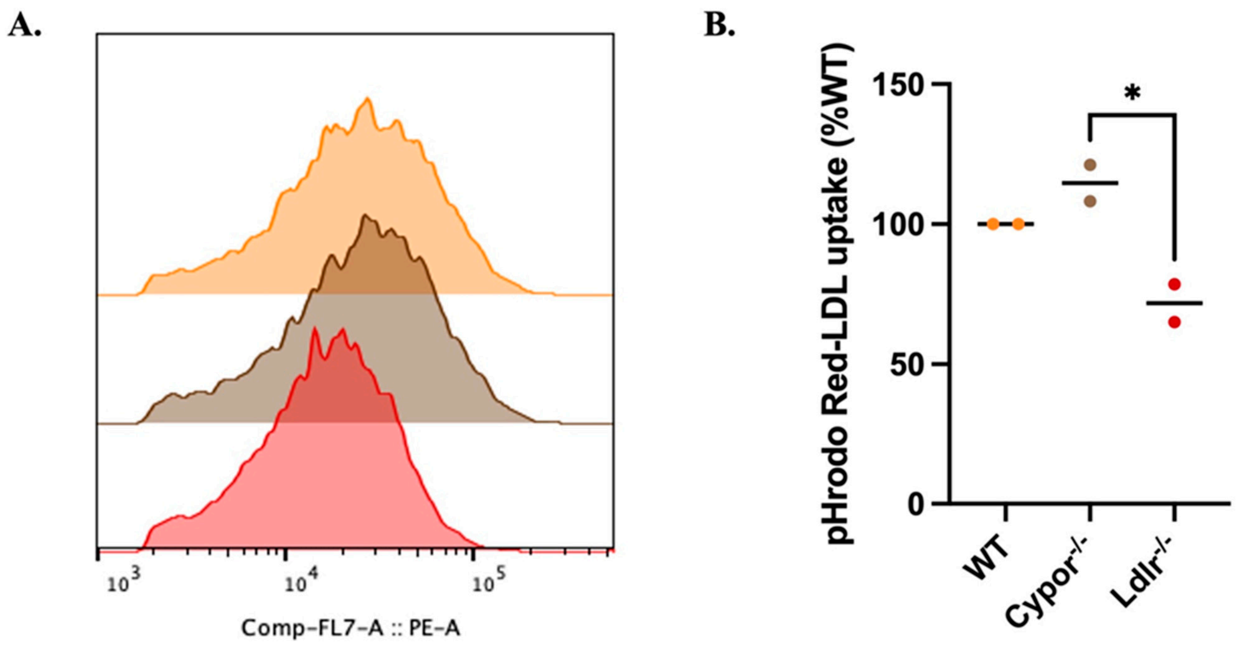

Supplementary Figure S2. Cypor-deficient hepatocytes show increased uptake compared to *Ldlr$^{-/-}$* hepatocytes. (A) Representative flow cytometry histogram from hepatocytes treated with pHrodo-Red labeled LDL-C conjugate. Colors of distributions correspond to respective groups in (B) LDL-C uptake in gene-edited *Cypor$^{-/-}$* and untransfected *Ldlr$^{-/-}$* hepatocytes relative to untransfected C57BL/6J (WT) control hepatocytes after 4-hour incubation in pHrodo-Red labeled LDL-C (n = 2). Bars represent the mean. Differences are not significant unless indicated. Levels of significance *P < 0.05 (one-way ANOVA with Tukey's multiple comparison).

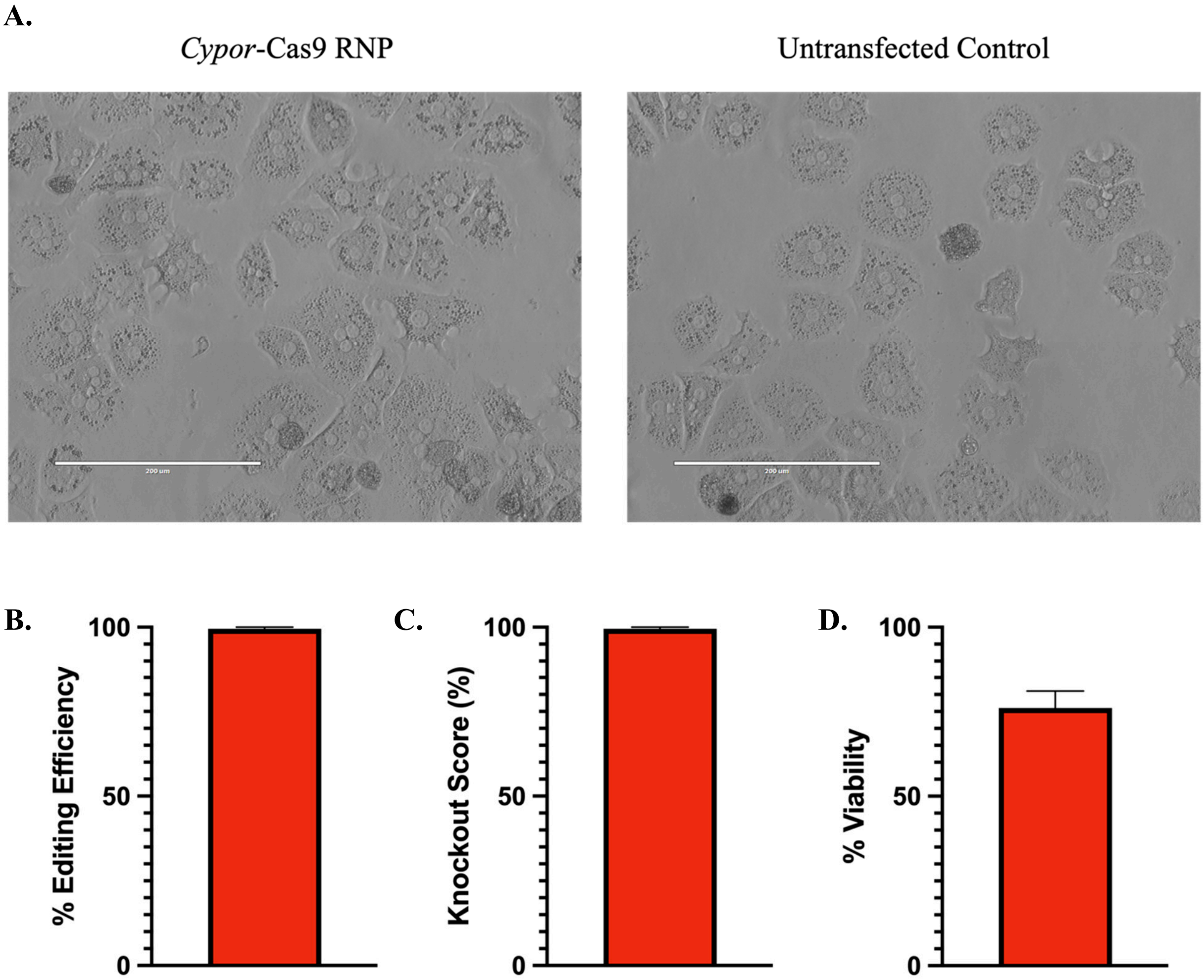

Supplementary Figure S3. ***In vitro*** **data from plated C57BL/6J donor hepatocytes used in APAP selection study.** (**A**) Phase contrast images taken 24 hours after electroporation. The scale bar represents 200 μm (**B**) The on-target indel frequency and (**C**) knockout score analyzed using the Synthego ICE CRISPR analysis tool. The mean indels and knockout score observed was at 99.5% for cells electroporated with *Cypor*-aiming Cas9 RNP. (**D**) Cell viability measured using MTT assay and normalized to the untransfected control cells. The mean viability observed as 76% for Cas9 RNP-treated cells. Error bars represent the SEM.

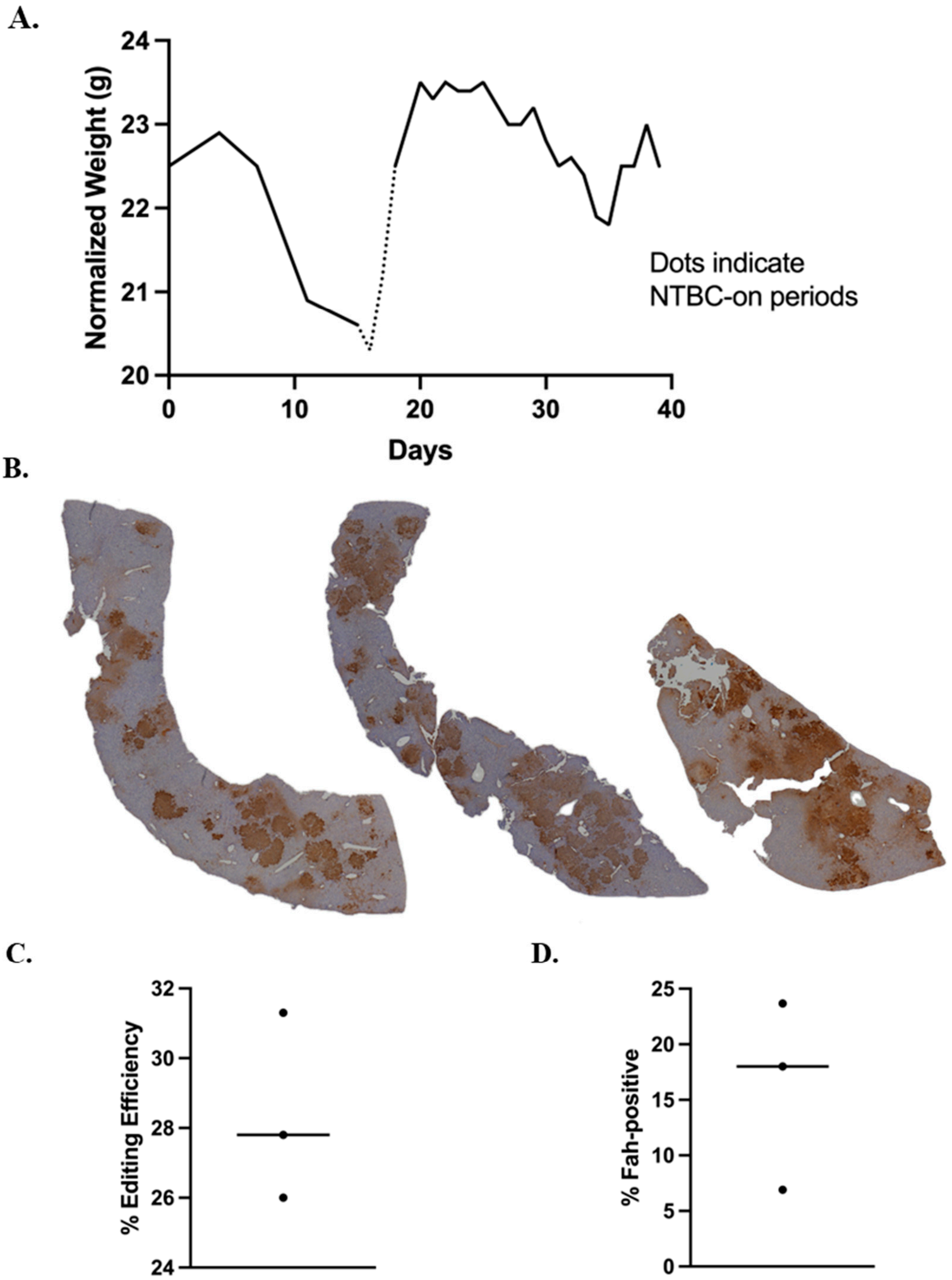

Supplementary Figure S4. Cypor-deficient hepatocytes transplanted into *Fah*$^{-/-}$ mice as engraftment controls for the APAP selection study. (A) Normalized weight data for recipient *Fah*$^{-/-}$ mouse (*n* =1) transplanted with Cypor-deficient hepatocytes. (**B**) IHC staining against Fah in liver tissue sections harvested at 40 days post-transplantation. (**C**) The on-target indel frequency measured using TIDE analysis in genomic DNA isolated from liver homogenates. (**D**) The % Fah-positive area measured using ImageJ analysis of Fah-stained IHC images. (*n* = 3 tissue replicates in B, C, and D).

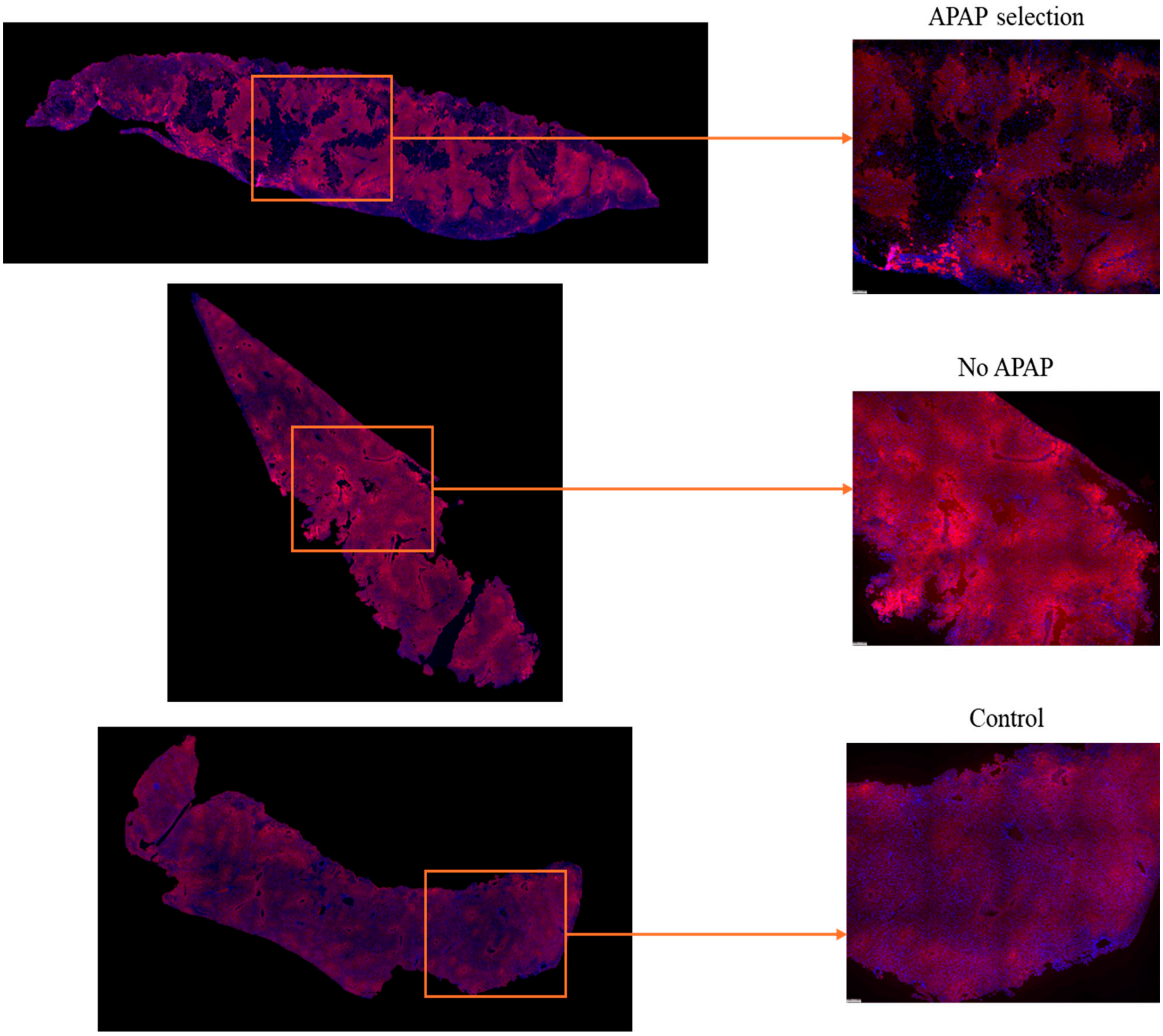

Supplementary Figure S5. Representative Cypor-IF stained liver tissue for *Ldlr*$^{-/-}$ recipient mice in the APAP selection study. Representative Cypor-IF stained liver tissue in APAP selected, no-APAP and untransplanted *Ldlr*$^{-/-}$ mice. Scale bar represents 100 µm.

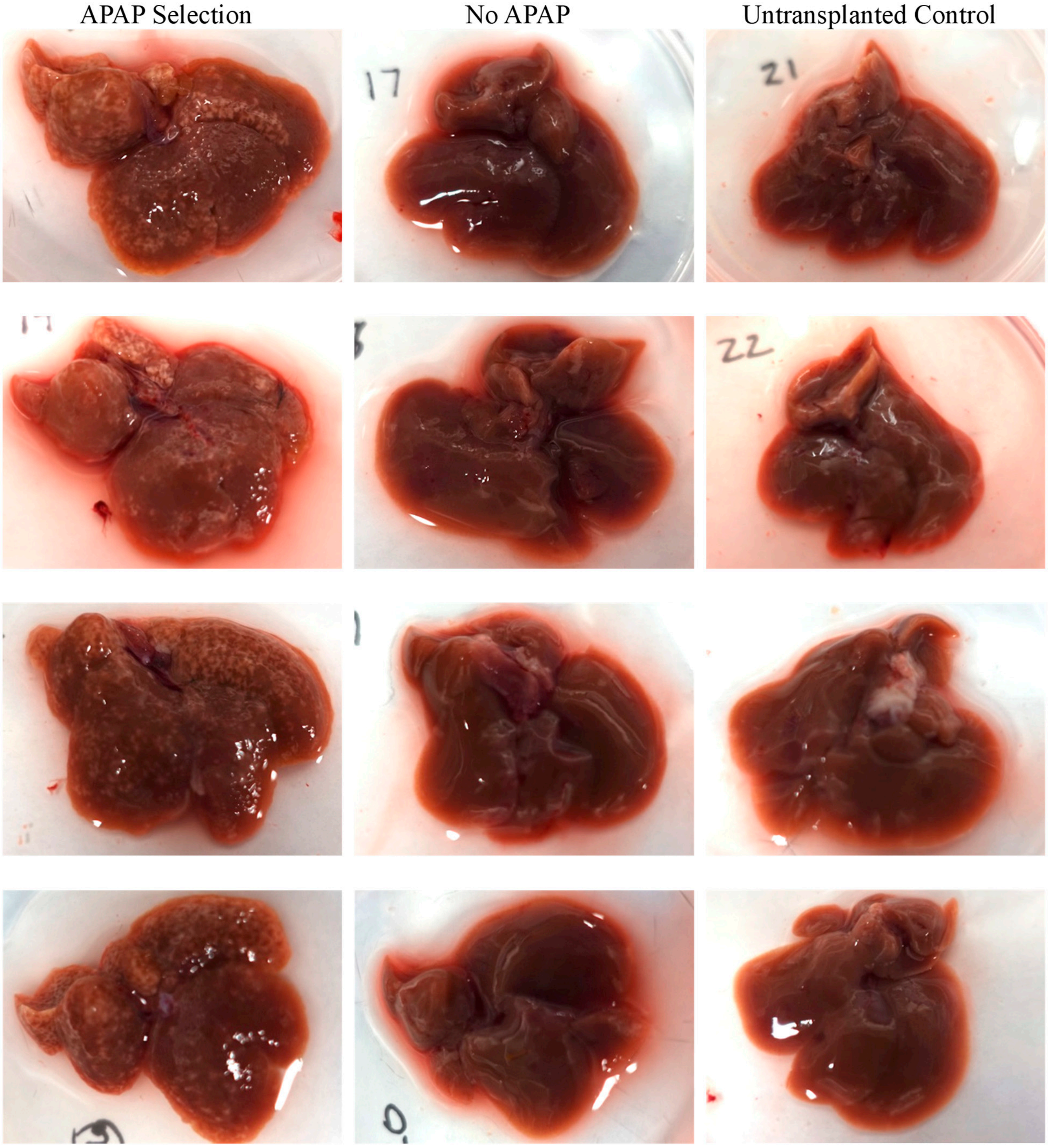

Supplementary Figure S6. Gross liver harvested from *Ldlr*$^{-/-}$ recipient mice in the APAP selection study. The first column shows liver harvested from recipient mice subjected to APAP selection. The second column shows liver images from transplanted mice without APAP selection. The third column shows livers harvested from untreated control mice. Images were taken immediately after mice were sacrificed.

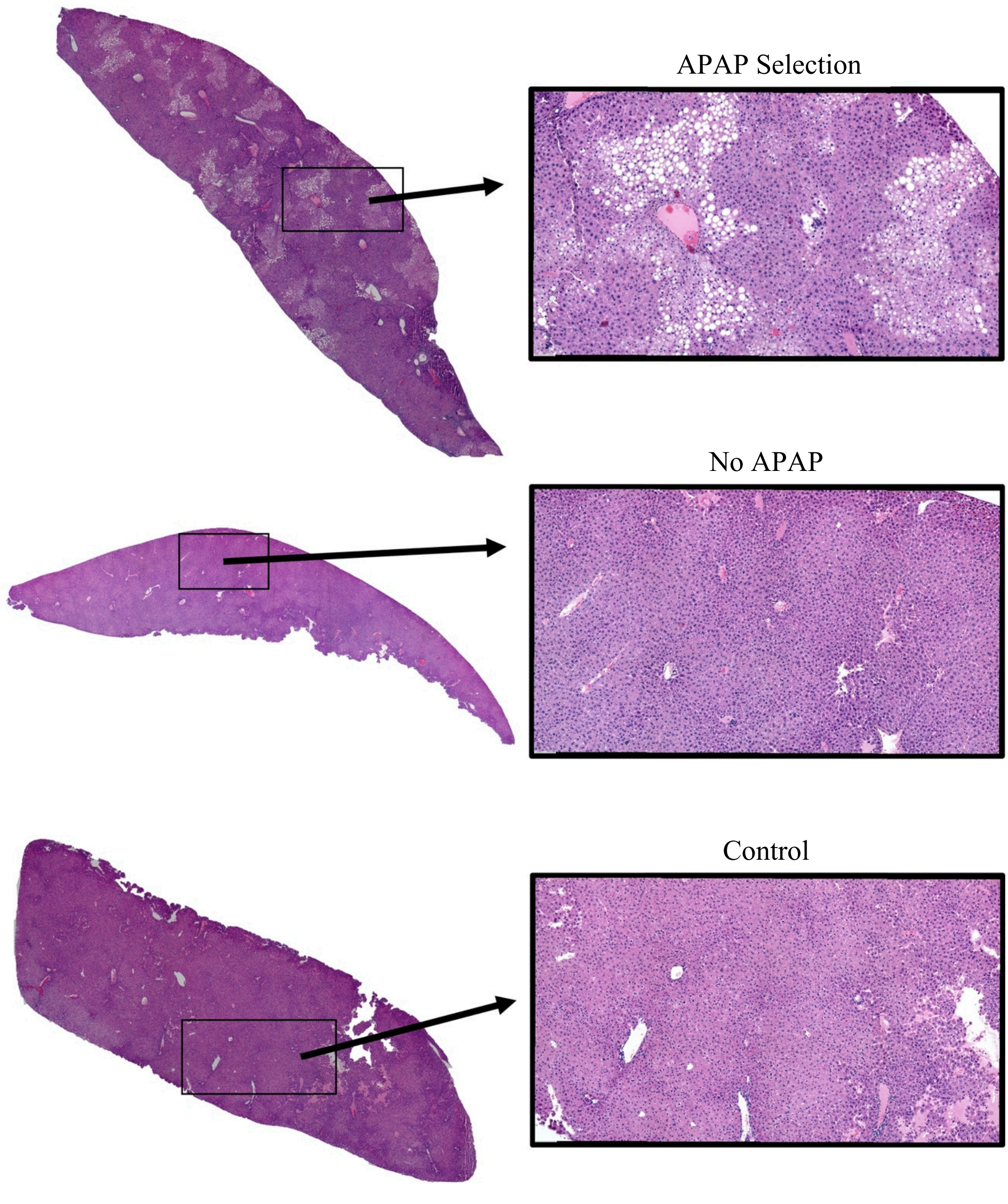

Supplementary Figure S7. Representative H&E-stained liver histology for *Ldlr$^{-/-}$* recipient mice in the APAP selection study. Representative image of H&E staining of left lateral lobe section from APAP selected, no-APAP and untransplanted *Ldlr$^{-/-}$* mice used for histological assessment. Scale bar represents 100 µm.

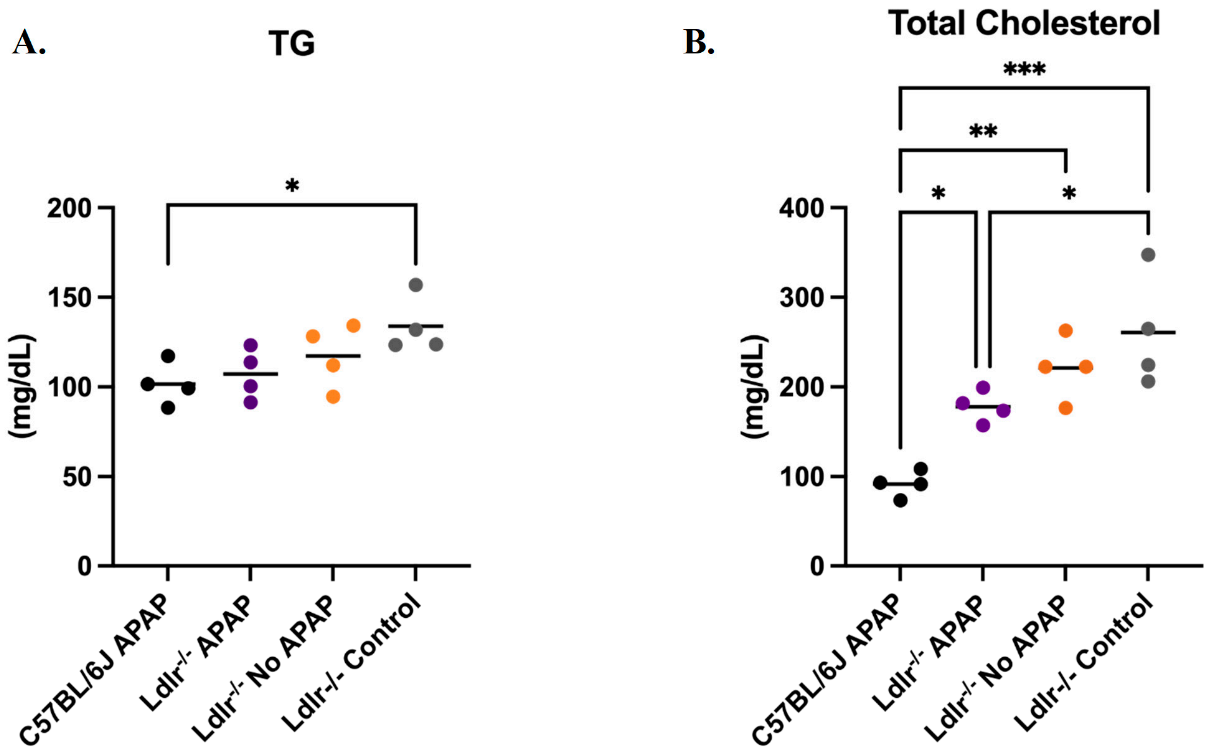

Supplementary Figure S8. Lipid levels in blood plasma after 12 weeks of APAP selection. (A) Plasma triglyceride (TG) and (**B**) total cholesterol levels were measured using Pointe Scientific assay kits. Blood samples were collected at the time of sacrifice from C57BL/6J mice and *Ldlr*$^{-/-}$ recipient mice ($n = 4$) transplanted with *Cypor*-deficient hepatocytes and subject to APAP selection. *Ldlr*$^{-/-}$ mice transplanted with *Cypor*-deficient hepatocytes receiving no APAP treatment and untreated *Ldlr*$^{-/-}$ mice were used as controls ($n = 4$). Error bars represent SEM. Differences are not significant unless indicated. Levels of significance, *P < 0.0332, **P < 0.0021, ***P < 0.0002 (one-way ANOVA with Tukey's multiple comparisons).

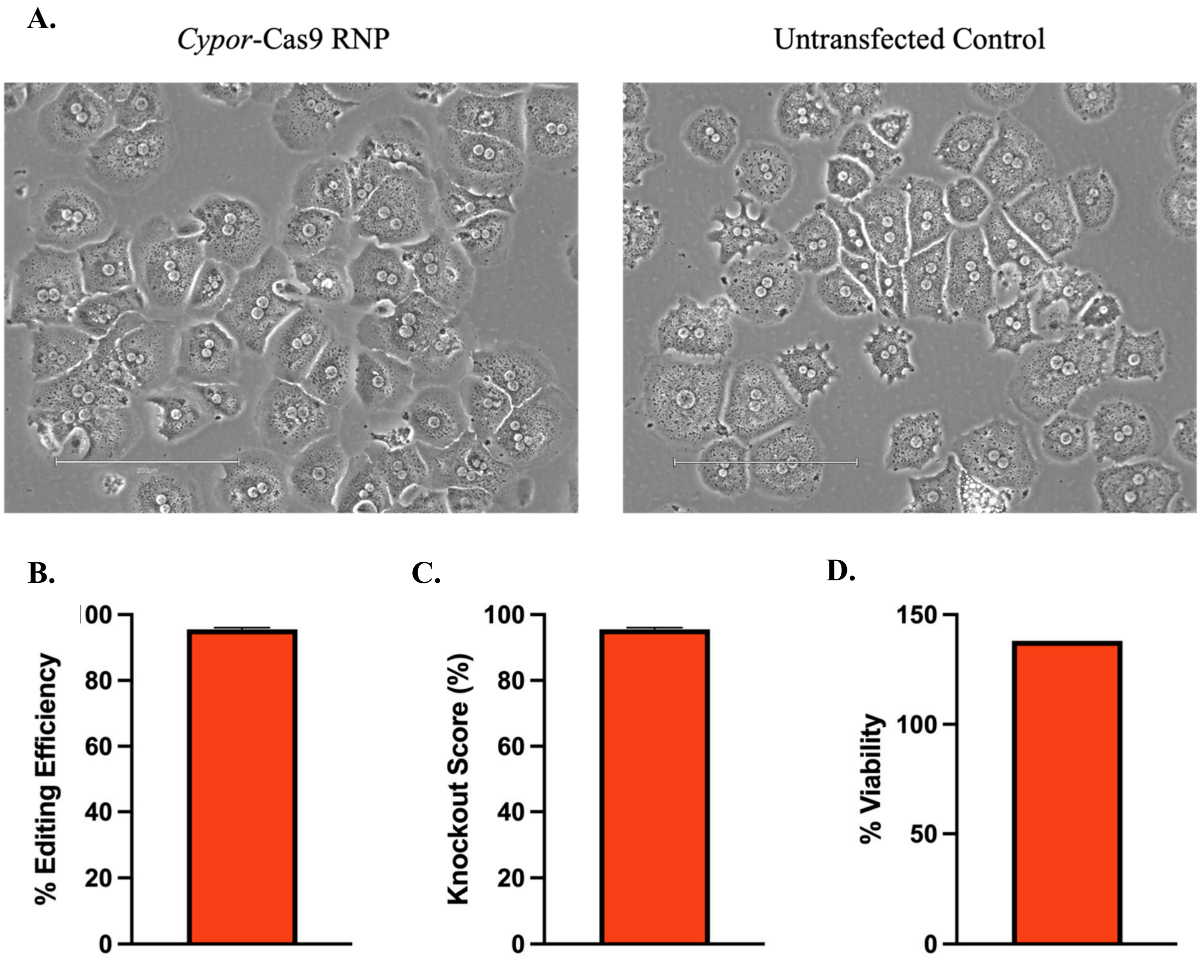

Supplementary Figure S9. ***In vitro*** **data from plated C57BL/6J donor hepatocytes used in APAP selection study with western diet.** (**A**) Phase contrast images taken at 24 hours after electroporating *Cypor*-Cas9 RNPs. (**B**) The on-target indels and (**C**) knockout score analyzed using Synthego ICE CRISPR analysis tool. (**D**) Cell viability was measured using MTT assay and normalized to the unedited control. Error bars represent the SEM.

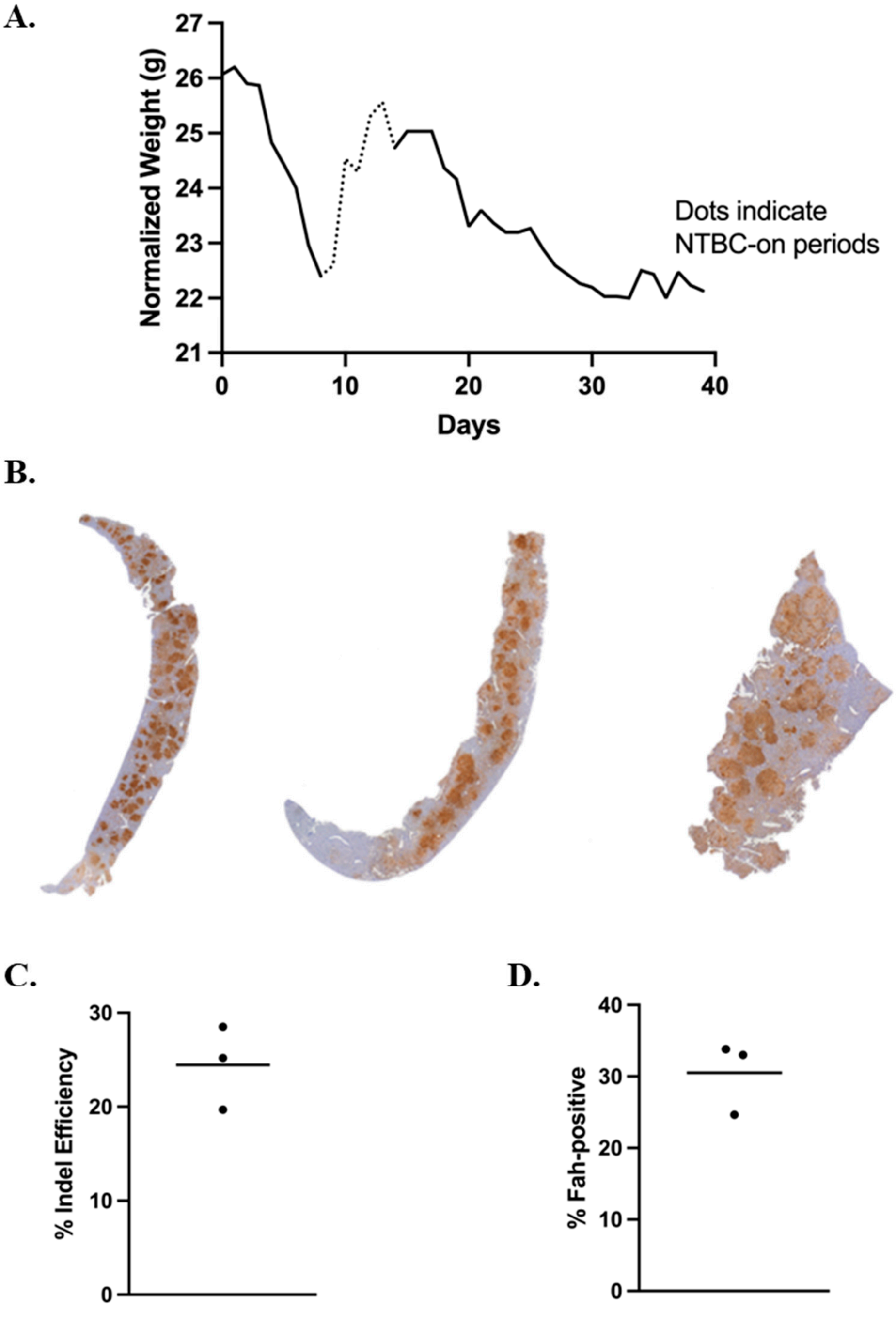

Supplementary Figure S10. Selection of Cypor-deficient hepatocytes in *Fah$^{-/-}$* mice via NTBC cycling as engraftment controls for APAP selection study with western diet. (A) Normalized weight data for *Fah$^{-/-}$* recipient mice transplanted with Cypor-deficient hepatocytes (*n* = 3). (**B**) Representative Fah-stained IHC images of fixed liver sections from *Fah$^{-/-}$* recipient mice. (**C**) On-target indels measured using TIDE analysis in DNA isolated from liver homogenates (*n* = 3) and (**D**) % Fah-positive area measured using ImageJ analysis of IHC stained slides. 3 slides per mouse and 3 biological replicates were used to quantify the percentage Fah-positive area.

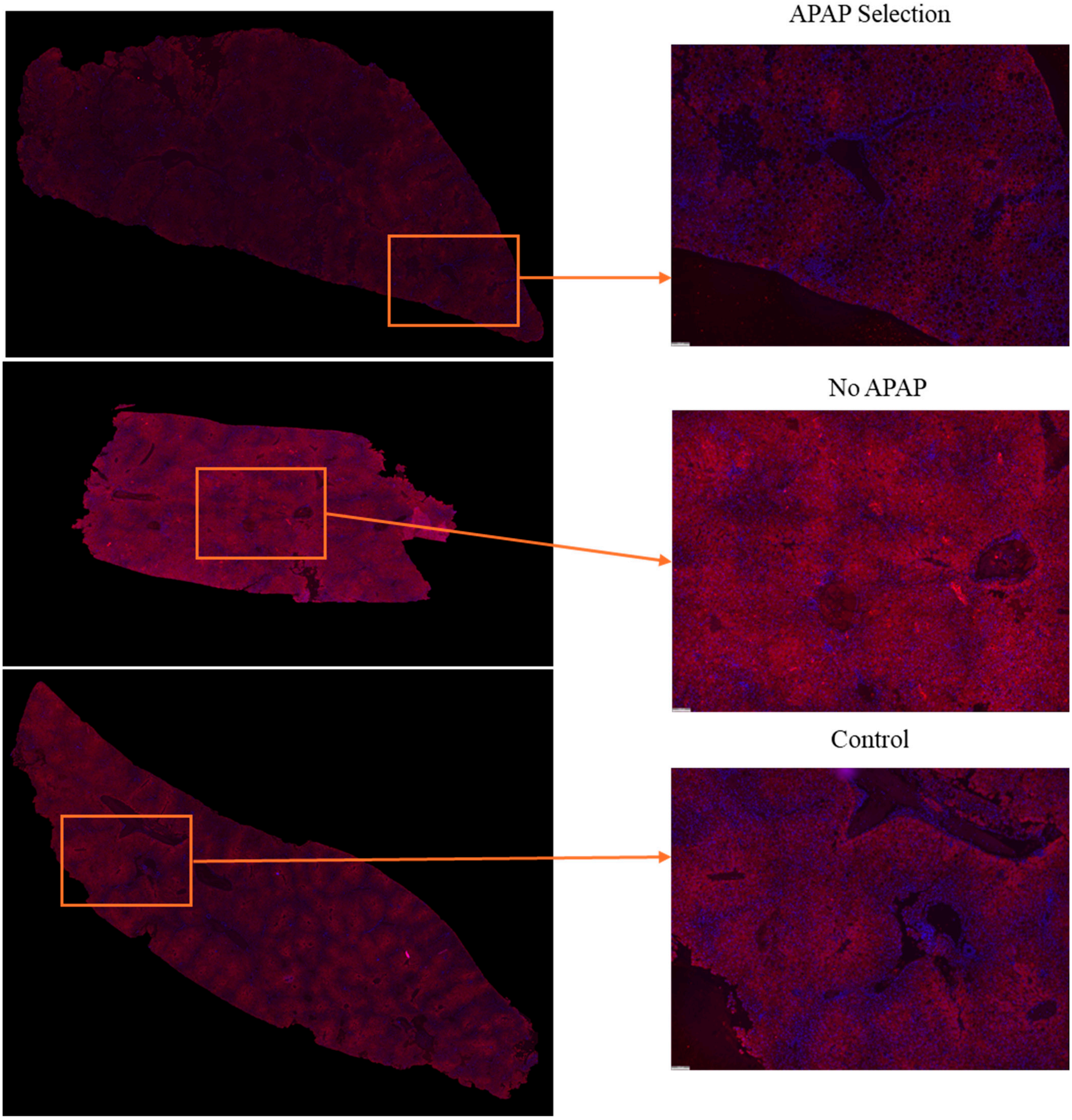

Supplementary Figure S11. Representative Cypor-IF stained liver histology for *Ldlr*$^{-/-}$ recipient mice in the APAP Western Diet selection study. Representative Cypor-IF stained liver tissue in APAP selected, no-APAP and untransplanted *Ldlr*$^{-/-}$ mice. Scale bar represents 100 µm.

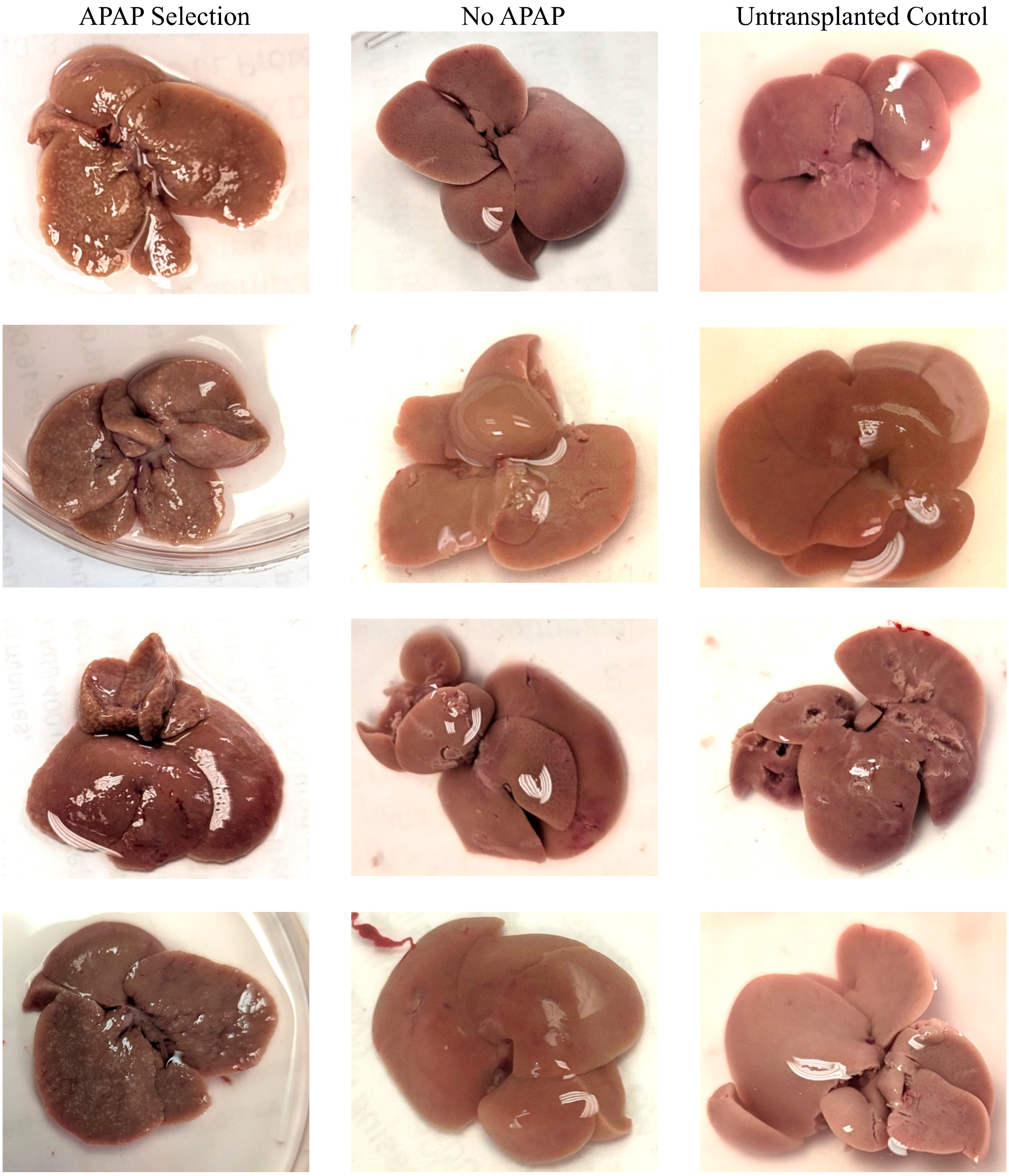

Supplementary Figure S12. Gross liver images of *Ldlr*$^{-/-}$ mice in APAP selection study with western diet. At 15 weeks after transplantation, mice were placed on a western diet for 12 weeks. The first column shows liver images for mice that were treated with APAP. The second column shows liver images from transplanted mice that did not receive APAP treatment. The third column shows liver images from untransplanted control mice without APAP treatment. Pictures were taken immediately after the mice were sacrificed.

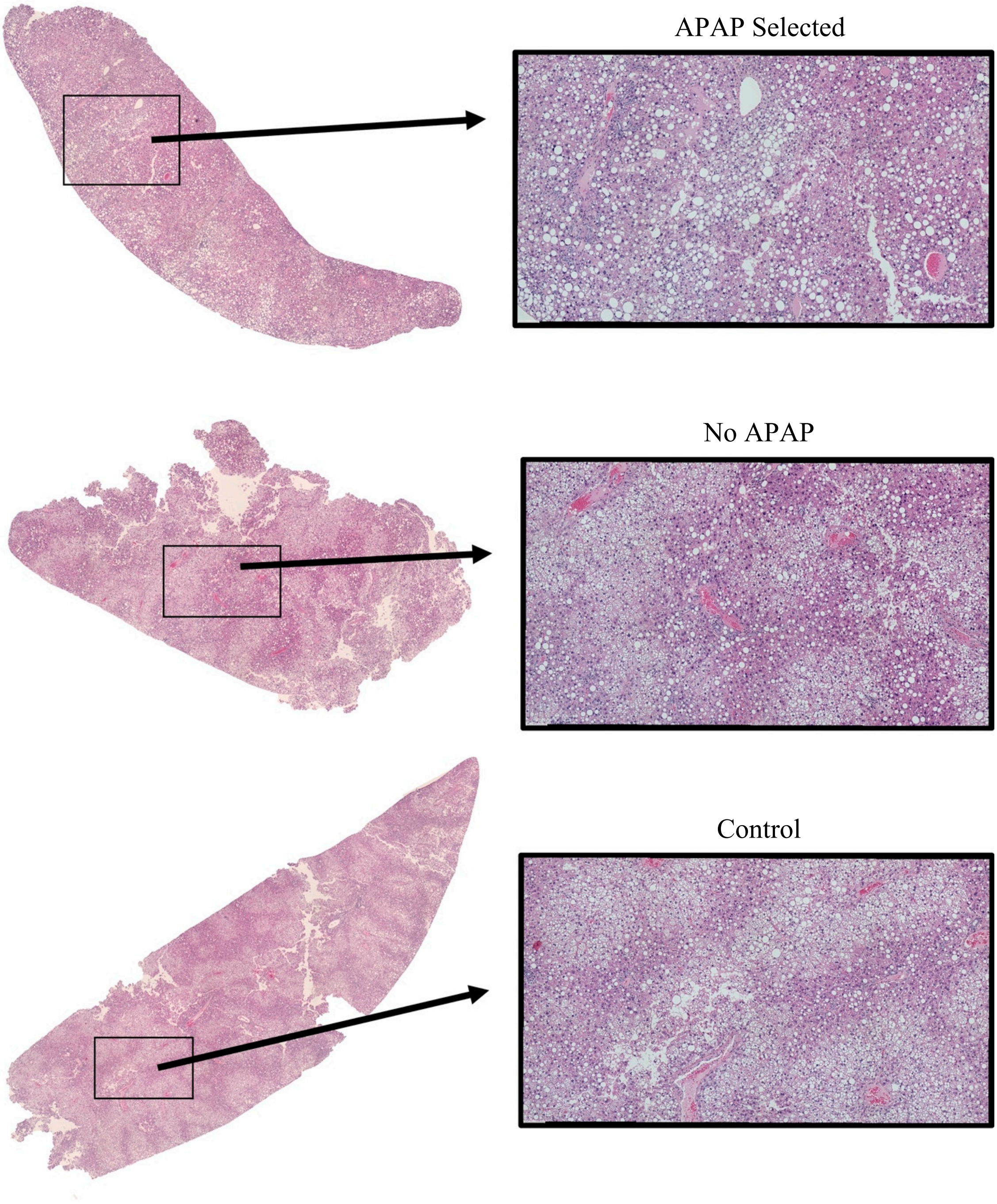

Supplementary Figure S13. Representative H&E-stained liver histology for *Ldlr*$^{-/-}$ recipient mice in the APAP selection study with western diet. Representative image of H&E staining of left lateral lobe section from APAP selected, no-APAP and untransplanted *Ldlr*$^{-/-}$ mice used for histological assessment. Scale bar represents 100 µm.

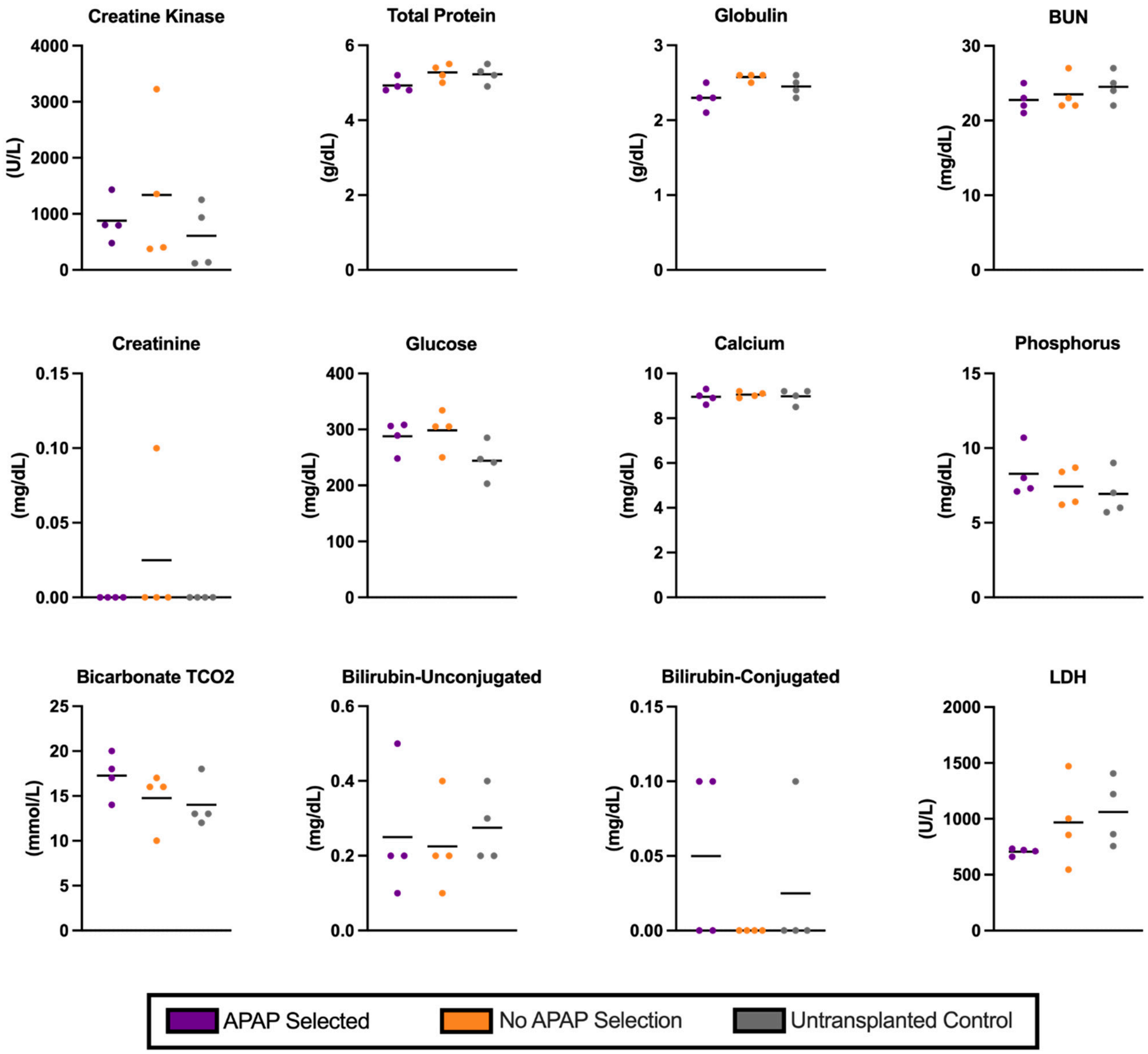

Supplementary Figure S14. Liver and kidney panels for *Ldlr*$^{-/-}$ mice subjected to APAP selection and placed on a western diet. Serum samples were collected at the time of sacrifice from *Ldlr*$^{-/-}$ mice transplanted with Cypor-deficient cells and treated with APAP. The controls consisted of *Ldlr*$^{-/-}$ transplanted mice with no APAP treatment and untreated *Ldlr*$^{-/-}$ mice ($n = 4$). Differences are not significant unless indicated. Levels of significance *$P < 0.01$ (one-way ANOVA with Tukey's multiple comparison).

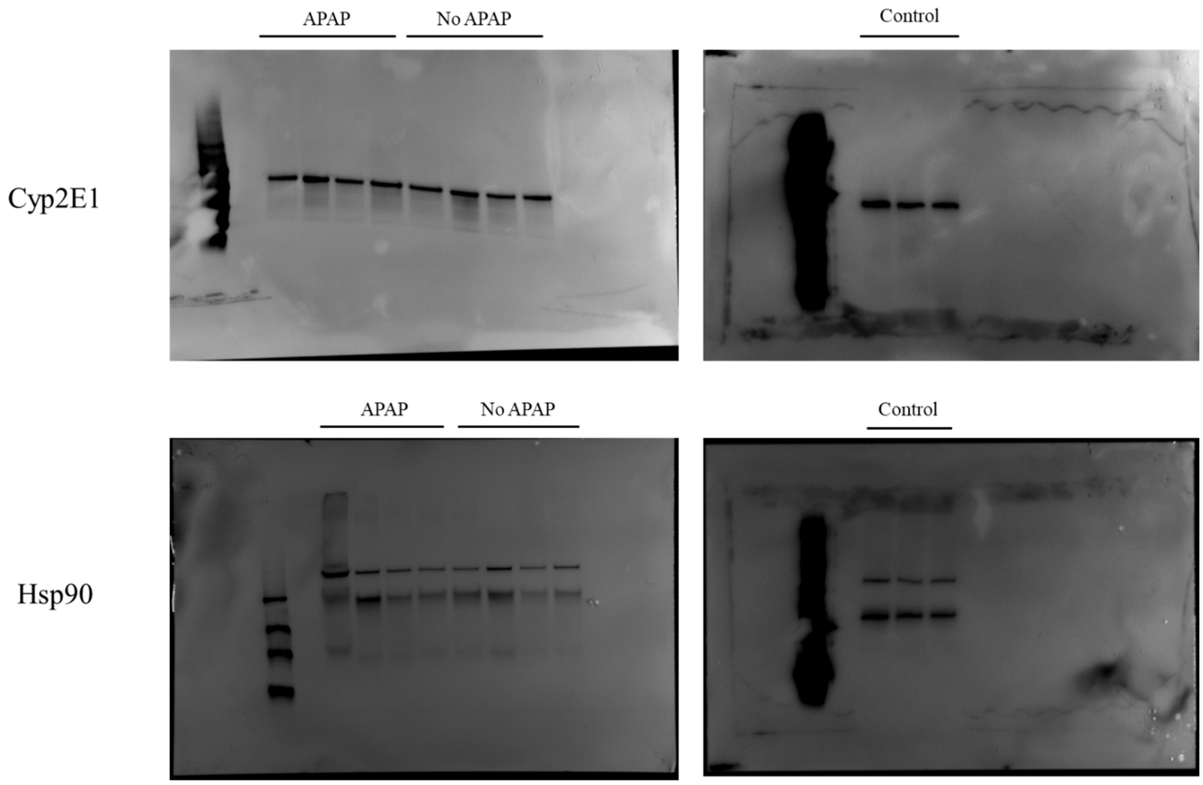

Supplementary Figure S15. Uncropped original immunoblot images corresponding to the data shown in main Figure 4A. Original images were used to determine expression of Cyp2E1 and Hsp90 in APAP-treated, no-APAP, and untransplanted control *Ldlr*$^{-/-}$ mice (n = 4).

Supplementary Table S1. List of mouse IDs and respective experimental condition and APAP dosage maintained throughout study.

Mouse ID	Experiment	Experimental Condition	Maintained APAP Dosage [mg/kg]
13	APAP Selection	APAP Injections	300
14	APAP Selection	APAP Injections	300
15	APAP Selection	APAP Injections	325
16	APAP Selection	APAP Injections	350
17	APAP Selection	No APAP	0
18	APAP Selection	No APAP	0
19	APAP Selection	No APAP	0
20	APAP Selection	No APAP	0
21	APAP Selection	Untransplanted Control	0
22	APAP Selection	Untransplanted Control	0
23	APAP Selection	Untransplanted Control	0
24	APAP Selection	Untransplanted Control	0
65	APAP Selection + Western Diet	APAP Injections	325
66	APAP Selection + Western Diet	APAP Injections	350
67	APAP Selection + Western Diet	APAP Injections	350
68	APAP Selection + Western Diet	APAP Injections	325
69	APAP Selection + Western Diet	No APAP	0
70	APAP Selection + Western Diet	No APAP	0
71	APAP Selection + Western Diet	No APAP	0
72	APAP Selection + Western Diet	No APAP	0
73	APAP Selection + Western Diet	Untransplanted Control	0
74	APAP Selection + Western Diet	Untransplanted Control	0
75	APAP Selection + Western Diet	Untransplanted Control	0
76	APAP Selection + Western Diet	Untransplanted Control	0

Supplementary Table S2. Histological assessment of H&E-stained histology images of the liver for APAP selection study.

Mouse ID	Experimental condition	H&E Histological assessment
13	LDLR-/- APAP Selection	Lipid filled nodules, 10-30%; many small basophilic cells perivascular, probably lymphocytes
14	LDLR-/- APAP Selection	10% lipid nodules, lots of inflammatory cells, several large lipid nodules
15	LDLR-/- APAP Selection	Lipid filled nodules; 30%, modest amount of inflammatory cells
16	LDLR-/- APAP Selection	Lipid filled nodules; 30-70%, modest amount of inflammatory cells
17	LDLR-/- No APAP	No nodules; normal liver morphology, minimal inflammation
18	LDLR-/- No APAP	No nodules; normal liver morphology, modest inflammation
19	LDLR-/- No APAP	No nodules; normal liver morphology, minimal inflammatory infiltrate
20	LDLR-/- No APAP	No nodules; normal liver morphology, minimal inflammatory infiltrate
21	Control	No nodules; normal liver morphology, minimal inflammatory infiltrate
22	Control	Normal

Supplementary Table S3. Histological assessment of H&E-stained histology images of the liver for APAP selection study with western diet.

Mouse ID	Experimental condition	H&E Histological assessment
65	LDLR-/- APAP Selection	Macrovesicular steatosis throughout; no clear nodules; looks like controls
66	LDLR-/- APAP Selection	Massive generalized steatosis; modest inflammation
67	LDLR-/- APAP Selection	Massive generalized steatosis; modest inflammation
68	LDLR-/- APAP Selection	Massive steatosis and inflammation
69	LDLR-/- No APAP	Admixture of areas with micro-vesicular and macrovesicular steatosis; massive generalized steatosis; modest inflammation
70	LDLR-/- No APAP	Admixture of areas with micro-vesicular and macrovesicular steatosis
71	LDLR-/- No APAP	Admixture of steatotic and normal hepatocytes ~ 50%, Looks zonal rather than nodular. Periportal: normal; pericentral: fatty
72	LDLR-/- No APAP	Admixture of steatotic and normal hepatocytes ~ 50%, Looks zonal rather than nodular. Periportal: normal; pericentral: fatty
73	Control	Modest steatosis; no nodules
74	Control	Normal liver; minimal steatosis
75	Control	Modest marcovesicular steatosis throughout
76	Control	Marcovesicular steatosis throughout
